# AI-Driven Diagnostic Assistance in Medical Inquiry: Reinforcement Learning Algorithm Development and Validation

**DOI:** 10.2196/54616

**Published:** 2024-08-23

**Authors:** Xuan Zou, Weijie He, Yu Huang, Yi Ouyang, Zhen Zhang, Yu Wu, Yongsheng Wu, Lili Feng, Sheng Wu, Mengqi Yang, Xuyan Chen, Yefeng Zheng, Rui Jiang, Ting Chen

**Affiliations:** 1 Shenzhen Center for Disease Control and Prevention Shenzhen China; 2 Department of Computer Science and Technology Tsinghua University Beijing China; 3 Institute of Artificial Intelligence Tsinghua University Beijing China; 4 Beijing National Research Center for Information Science and Technology Tsinghua University Beijing China; 5 Jarvis Research Center Tencent YouTu Lab Shenzhen China; 6 Beijing Tsinghua Changgung Hospital School of Clinical Medicine Tsinghua University Beijing China; 7 Tencent Healthcare Shenzhen China; 8 Department of Automation Tsinghua University Beijing China

**Keywords:** inquiry and diagnosis, electronic health record, reinforcement learning, natural language processing, artificial intelligence

## Abstract

**Background:**

For medical diagnosis, clinicians typically begin with a patient’s chief concerns, followed by questions about symptoms and medical history, physical examinations, and requests for necessary auxiliary examinations to gather comprehensive medical information. This complex medical investigation process has yet to be modeled by existing artificial intelligence (AI) methodologies.

**Objective:**

The aim of this study was to develop an AI-driven medical inquiry assistant for clinical diagnosis that provides inquiry recommendations by simulating clinicians’ medical investigating logic via reinforcement learning.

**Methods:**

We compiled multicenter, deidentified outpatient electronic health records from 76 hospitals in Shenzhen, China, spanning the period from July to November 2021. These records consisted of both unstructured textual information and structured laboratory test results. We first performed feature extraction and standardization using natural language processing techniques and then used a reinforcement learning actor-critic framework to explore the rational and effective inquiry logic. To align the inquiry process with actual clinical practice, we segmented the inquiry into 4 stages: inquiring about symptoms and medical history, conducting physical examinations, requesting auxiliary examinations, and terminating the inquiry with a diagnosis. External validation was conducted to validate the inquiry logic of the AI model.

**Results:**

This study focused on 2 retrospective inquiry-and-diagnosis tasks in the emergency and pediatrics departments. The emergency departments provided records of 339,020 consultations including mainly children (median age 5.2, IQR 2.6-26.1 years) with various types of upper respiratory tract infections (250,638/339,020, 73.93%). The pediatrics department provided records of 561,659 consultations, mainly of children (median age 3.8, IQR 2.0-5.7 years) with various types of upper respiratory tract infections (498,408/561,659, 88.73%). When conducting its own inquiries in both scenarios, the AI model demonstrated high diagnostic performance, with areas under the receiver operating characteristic curve of 0.955 (95% CI 0.953-0.956) and 0.943 (95% CI 0.941-0.944), respectively. When the AI model was used in a simulated collaboration with physicians, it notably reduced the average number of physicians’ inquiries to 46% (6.037/13.26; 95% CI 6.009-6.064) and 43% (6.245/14.364; 95% CI 6.225-6.269) while achieving areas under the receiver operating characteristic curve of 0.972 (95% CI 0.970-0.973) and 0.968 (95% CI 0.967-0.969) in the scenarios. External validation revealed a normalized Kendall τ distance of 0.323 (95% CI 0.301-0.346), indicating the inquiry consistency of the AI model with physicians.

**Conclusions:**

This retrospective analysis of predominantly respiratory pediatric presentations in emergency and pediatrics departments demonstrated that an AI-driven diagnostic assistant had high diagnostic performance both in stand-alone use and in simulated collaboration with clinicians. Its investigation process was found to be consistent with the clinicians’ medical investigation logic. These findings highlight the diagnostic assistant’s promise in assisting the decision-making processes of health care professionals.

## Introduction

### Background

The growing demand for intelligent clinical decision support systems (CDSSs) has become increasingly evident in the health care landscape today [1,2]. The surge in demand can be attributed to advances in medical knowledge, the accumulation of health care data, and the rapid progression of artificial intelligence (AI) and machine learning technologies. Traditionally, health care professionals have relied heavily on their individual experiences and medical expertise to make clinical decisions, which are often susceptible to subjective biases and information gaps [3,4]. Furthermore, the world faces challenges related to insufficient medical resources, particularly in regions where a shortage of health care professionals impedes timely access to care. Consequently, intelligent CDSSs have been developed with the potential to enhance patient care quality, reduce medical costs, and minimize diagnostic errors. These systems were developed by analyzing and training data from electronic health records (EHRs) and medical literature [5,6] using the power of extensive data analysis, machine learning algorithms, and natural language processing techniques.

Our research is focused on advancing CDSSs with a primary emphasis on aiding the diagnostic process before the diagnosis is made, specifically during the information-gathering phase. Existing diagnostic support systems often rely on comprehensive patient information, such as EHRs and medical images [7-13], to provide diagnostic predictions only at the final step of the medical investigation process. Thus, the vital need for decision support during the intermediate steps of the diagnostic process is largely overlooked. The clinical diagnostic process is a complex procedure that involves a series of inquiries and examinations. In this dynamic process, health care professionals continuously gather information, adjust hypotheses, and refine diagnostic reasoning until the optimal diagnosis and treatment are determined. The process is not merely a sequence of isolated steps but a dynamic and evolving interaction between the clinician and the patient. Conventional diagnostic support systems tend to provide little to no guidance during these intermediate steps, falling short of providing meaningful support when it is most needed.

A variety of automatic disease diagnosis techniques have emerged recently as a result of the advancement of AI with the goal of assisting in the middle stages of clinical decision-making [14-21]. However, a common limitation among these methods is their exclusive focus on online disease diagnosis. Online health care platforms have undoubtedly revolutionized medical consultations by facilitating remote access to health care professionals. Patients can seek advice and preliminary assessments for various ailments without visiting a health care facility physically. Unfortunately, there are inherent constraints to these platforms. Online consultations are primarily reliant on textual descriptions, making it challenging to gather essential information that requires palpation, auscultation, or specialized laboratory investigations, which collectively form the foundation of a comprehensive and accurate diagnosis.

Large language models (LLMs) such as ChatGPT [22] have demonstrated remarkable proficiency in following instructions and generating humanlike responses across various domains. LLMs have already attracted a lot of attention regarding their potential applications in health care settings, which include facilitating clinical documentation, summarizing research papers, assisting with medical education [23], or acting as a chatbot to respond to questions from patients about their specific concerns [24,25]. While LLMs have access to extremely large corpora containing medical dialogues, knowledge bases, and the literature, there is a significant gap in access to large-scale real patient records [26]. This lack of actual clinical data significantly impacts their ability to provide tailored inquiry recommendations for patients in varying clinical scenarios. In addition, ethical [24,25] and security [27] concerns further complicate the incorporation of sensitive health records into the training of LLMs.

### Objectives

To address the gap in diagnostic decision support, we present MedRIA, a medical reinforcement learning inquiry assistant. MedRIA is designed to facilitate the medical investigation process by intelligently guiding inquiries; symptom assessments; examination recommendations; and, in particular, the transitions between them. To the best of our knowledge, no previous system has successfully managed the entire diagnostic process. MedRIA learns its inquiry capabilities from a large number of processed EHRs. MedRIA uses a reinforcement learning actor-critic framework [28] to explore rational and effective inquiry logic. Following the standard inquiry process of physicians [29], we segmented the inquiry into 4 stages: inquiring about symptoms and medical history, conducting physical examinations (PEs), requesting auxiliary examinations (AEs), and terminating the inquiry with a diagnosis. MedRIA’s ability to tailor inquiry strategies according to patient-specific conditions positions it as a promising solution for regions or populations facing constraints in medical resources.

## Methods

### Processed Outpatient EHRs

**Figure 1:**
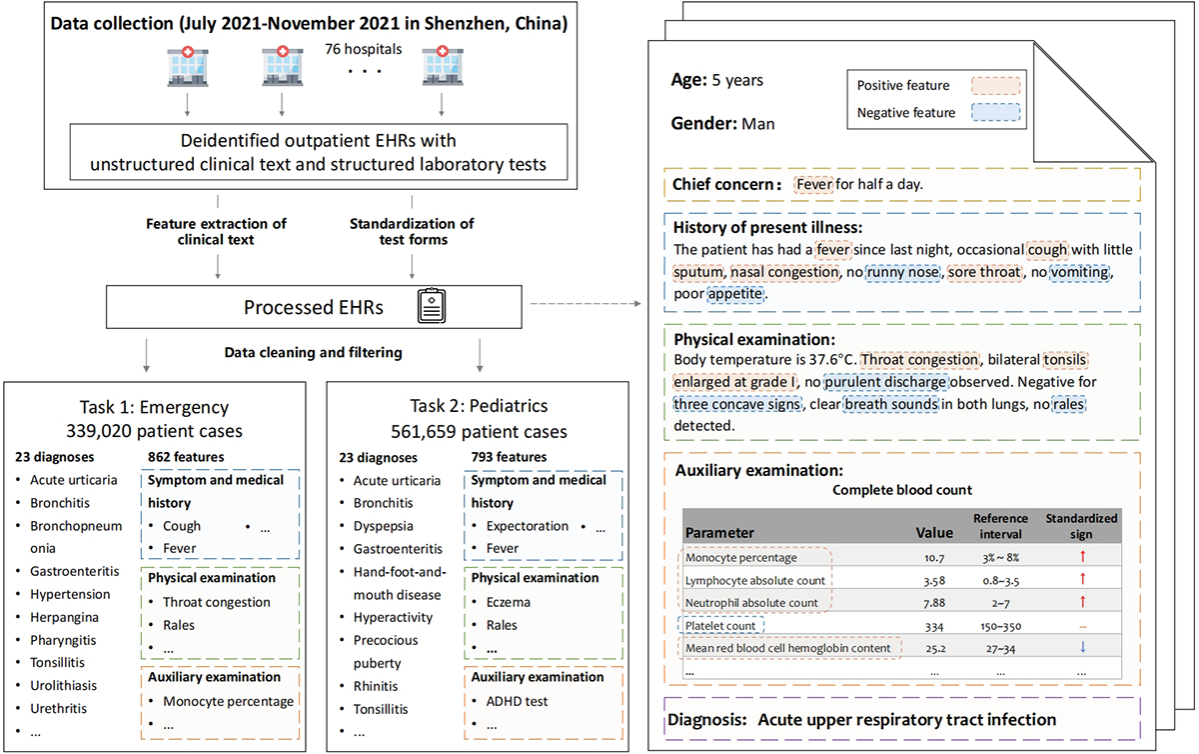
Schematic illustration of the data collection, data processing, and task building process for the development of MedRIA. ADHD: attention-deficit/hyperactivity disorder; EHR: electronic health record.

The Shenzhen Center for Disease Control and Prevention in Guangdong Province, China, collected deidentified outpatient EHRs from 76 hospitals in Shenzhen. We selected records of the emergency and pediatrics departments from July 2021 to November 2021. As depicted in Figure 1, these records consist of both unstructured textual information and structured laboratory test results, which underwent feature extraction and standardization using natural language processing techniques. An example of a processed EHR is provided on the right side of Figure 1.

By combining established medical terminologies with domain-specific knowledge from health care professionals, we constructed a comprehensive feature set for the feature extraction process. Initially, we used existing Chinese versions of general medical terminologies, including the *International Classification of Diseases, 10th Revision*, and Systematized Nomenclature of Medicine–Clinical Terms. In addition, we incorporated domain expertise by consulting with health care experts to identify relevant clinical concepts and terminology to the context of our study. The feature sets of the emergency and pediatrics EHRs are presented in Multimedia Appendices 1 and 2, respectively.

As depicted in Figure 1, after extracting features from clinical text and standardizing test forms, each record was converted into a feature set, where each feature was categorized into 1 of 3 types based on its source—symptom and medical history (SMH), PE, and AE—and assigned a value based on its data type. Symptom features (eg, *cough*) and some features observed from examinations (eg, *throat congestion* and *proteinuria*) were assigned binary values indicating whether the patient exhibited that feature. Numerical laboratory test features with specific reference ranges, such as *platelet count*, were categorized as high, low, or within the reference range. The values of descriptive features depend on their meanings. For example, *urine color* was given values such as *yellow*, *colorless*, *white*, *red*, and so on.

In our study, we used the Medical Bidirectional Encoder Representations From Transformers (MedBERT) model for feature extraction on clinical text, which was based on Bidirectional Encoder Representations From Transformers [30]. Using a vast corpus of Chinese clinical text data, including medical textbooks, online consultations, journal article abstracts, and deidentified EHRs, we pretrained the Bidirectional Encoder Representations From Transformers model to obtain MedBERT. We manually constructed 2 labeled data sets to fine-tune MedBERT to perform medical named entity recognition and entity normalization. In our pipeline, we first used fine-tuned MedBERT for medical named entity recognition on clinical text. Next, we proceeded to normalize the identified entities to our predefined feature set using the fine-tuned MedBERT for entity normalization. Finally, we used a string-matching negation detection mechanism to determine the values of binary features based on the contents of the text. For structured laboratory test results, we used a string-matching algorithm to standardize the test items to our predefined feature set. The feature values could be directly obtained from the structured results.

Following the feature extraction process, we conducted data cleaning and filtering. Details are presented in Multimedia Appendix 3 [31-37]. Finally, we filtered the data sets by department, creating 2 retrospective inquiry-and-diagnosis evaluation tasks for emergency and pediatrics. Emergency and pediatrics are the 2 busiest departments in hospitals and often put tremendous pressure on health care personnel. These departments require effective decision support tools to assist health care professionals in managing the workload efficiently while providing timely and accurate patient care.

### Reinforcement Learning Formulation

MedRIA takes as initial input the patient’s basic demographic information, including gender and age, along with features extracted from the patient’s chief concerns. Subsequently, MedRIA provides recommendations for the physician to inquire about specific features. As the physician conducts the inquiry and gathers patient information, MedRIA updates the patient’s status and continues to output the next relevant feature or suggests terminating the inquiry and providing a predicted diagnosis in terms of the probability distribution over potential disease diagnoses. This can be described as a Markov decision process [38], represented by the tuple *M*(*S*, *A*, *T*, *R*, *γ*). Here, *S* (state) is a set of vectors representing the inquiry states that incorporate both observed and unobserved features so far, denoted as x_O_ and x_U_, respectively. *A* (action) involves the actions available to MedRIA, including recommending specific features or suggesting the termination of the inquiry process. The goal is to determine the optimal inquiry strategy composed of a series of actions, denoted as π_θ_. At each step t, the policy π_θ_(a_t_|s_t_) determines the action a_t_ based on the current state s_t_. The transition function *T* represents the probability distribution of the next state s_t+1_ given the current state s_t_ and action a_t_, denoted as p(s_t+1_|s_t_, a_t_). *R* is a reward function used to assess the benefit of the transition (s_t_, a_t_, s_t+1_). *γ* is the discount factor for accumulating rewards at each step. The state-value function that reinforcement learning maximizes is the expected sum of discounted rewards given the policy π_θ_ and the state s_t_.

### Actor-Critic Framework

MedRIA uses the classic reinforcement learning actor-critic framework [28] involving 2 neural networks: the actor and the critic. The actor network determines the next action to take, whereas the critic network is responsible for providing feedback to the actor, evaluating the quality of actions and suggesting adjustments. On the basis of our previous work [39], we used a pretrained variational autoencoder (VAE) [31] as the backbone of the actor network. We used a supervised diagnostic prediction model, denoted as *D*, to provide the current disease prediction probability distribution *D*(s_t_) based on the state s_t_ to help decide on each inquiry action.

The actor of MedRIA makes decisions for the next action based on the observed features x_O_. It is intuitive to prioritize asking about feature x that has a high conditional probability p(x|x_O_). We leveraged the VAE to incorporate the conditional probability distribution between observed features x_O_ and unobserved features x_U_. A VAE defines a generative model of the form p(x, z)=∏_i_ p_Θ_(x_i_|z)p(z), where features x are generated from latent variables z. The number of dimensions of z is 64. p(z) represents a prior, often a spherical Gaussian distribution. p_Θ_(x|z) is represented by a 4-layer multilayer perceptron (MLP) decoder with parameters Θ. The VAE uses another neural network encoder with parameters Φ to generate the variational approximation of the posterior q_Φ_(z|x). To obtain p(x_U_|x_O_), we sampled ẑ from the VAE encoder as ẑ~q_Φ_(z|x) and then sampled x_U_ from the VAE decoder as p_Θ_(x_U_|ẑ). Let e_i_ represent the embedding vector for the ith observed feature x_i_, and let c_i_=[x_i_, e_i_] denote the concatenated input carrying information for x_i_. The number of dimensions of e_i_ is 64. Then, we used a 4-layer MLP to map the input c_i_ to a Gaussian distribution in latent space, with mean vector μ_i_ and variance vector V_i_. To address arbitrary partial observations of features during the inquiry process, we used a product-of-experts mechanism [40] to calculate the approximate posterior. The VAE was incorporated within the actor network to leverage the conditional probability distribution p(x_U_|x_O_). To obtain the action a_t_, x_O_ were first fed into the nested VAE, yielding decoded features that contain predictive information regarding x_U_. The decoded features were then concatenated with the current diagnostic confidence *D*(s_t_) and the representation of the current timestep n_t_. Here, n_t_=t / N_T_ represents the ratio of the number of completed inquiries t over a predefined maximum action count N_T_. N_T_ was set to 21 and 26 for the emergency and pediatrics tasks, respectively. Finally, a 2-layer MLP with softmax activation was used to map the concatenated vector to the action space.

The objective of the critic is to estimate the state-value function to optimize the policy π_θ_. Similar to the approach used in the actor, we concatenated the current state s_t_ with *D*(s_t_) and n_t_ to form an input vector to aid in estimation. In addition, we obtained the informative latent variable z_t_ by feeding observed features x_O_ into the VAE encoder and appended z_t_ to the input vector. Ultimately, a 5-layer MLP was used to map the input vector to predict the state value.

### Reward Shaping

The reward function for evaluating the gain in state transition plays a crucial role in the reinforcement learning process. We start by defining the short-term reward function *R*_short_ when the action is to inquire about a specific feature x. For MedRIA, the inquiry states can be categorized into 4 ordered stages: SMH (S_SMH_), PE (S_PE_), AE (S_AE_), and termination with diagnosis (S_TD_). During the inquiry process, there can be multiple states that fall under each stage. For example, the initial state of MedRIA falls under S_SMH_, followed by a sequence of states that fall under S_PE_, and so on. Correspondingly, all features are categorized according to the 3 inquiry stages S_SMH_, S_PE_, and S_AE_. When MedRIA selects a feature that falls under a particular inquiry stage, it signifies a transition from the inquiry stage to that stage. We ensure that MedRIA’s inquiry process adheres to the sequential nature of clinical inquiries, where features from previous stages are not considered for selection once the inquiry progresses to a subsequent stage. This is achieved by manually setting the probabilities of actions associated with features from previous stages to 0 in the action probability distribution of the actor.

To measure the consistency of MedRIA’s inquiries with those made by physicians, we introduce 3 functions: M_SMH_(s_t_), M_PE_(s_t_), and M_AE_(s_t_). These functions quantify the number of features that physicians have inquired about in the current state s_t_ but that MedRIA has not investigated. Specifically, M_SMH_(s_t_), M_PE_(s_t_), and M_AE_(s_t_) quantify the number of SMH features, PE features, and AE features, respectively. When calculating M_AE_(s_t_), all features within a laboratory test are considered as a unit. Essentially, M_AE_(s_t_) calculates the number of AEs that physicians have recommended in the current state s_t_ but that MedRIA has omitted. Correspondingly, when MedRIA selects a feature associated with an AE, it indicates that MedRIA has recommended the entire examination.

To assess the diagnostic quality of inquiries, we define a function Diff(s_t_, x, s_t+1_) to estimate the differential effect of querying the feature x in the transition from state s_t_ to state s_t+1_. If x belongs to the extracted features, meaning that the physician also inquired about that feature from the patient, then Diff(s_t_, x, s_t+1_) is defined as Diff(s_t_, x, s_t+1_)=|D_KL_[y||*D*(s_t_)]-D_KL_[y||*D*(s_t+1_)]|, where D_KL_ is the Kullback-Leibler divergence between 2 distributions and y is a one-hot vector indicating the final diagnosis made by the physician. The absolute difference between these 2 Kullback-Leibler divergences represents the degree to which the feature x influences the diagnosis results, with the final diagnosis made by the physician as the reference. If x does not belong to the extracted features, then Diff(s_t_, x, s_t+1_) is simply set to 0. Subsequently, we define the short-term reward function *R*_short_(s_t_, x, s_t+1_) as follows: when s_t_ belongs to S_SMH_ and s_t+1_ belongs to S_PE_, *R*_short_(s_t_, x, s_t+1_)=Diff(s_t_, x, s_t+1_)+α·I_phy_(x)-m·M_SMH_(s_t_); when s_t_ belongs to S_SMH_ and s_t+1_ belongs to S_AE_, *R*_short_(s_t_, x, s_t+1_)=Diff(s_t_, x, s_t+1_)+α·I_phy_(x)-m·(M_SMH_(s_t_)+M_PE_(s_t_)); when s_t_ belongs to S_PE_ and s_t+1_ belongs to S_AE,_ *R*_short_(s_t_, x, s_t+1_)=Diff(s_t_, x, s_t+1_)+α·I_phy_(x)-m·M_AE_(s_t_); otherwise, *R*_short_(s_t_, x, s_t+1_)=Diff(s_t_, x, s_t+1_)+α·I_phy_(x). I_phy_(x) is an indicator representing whether feature x belongs to the extracted features. If yes, it takes the value of 1; otherwise, it is 0. α and m are small constant factors to balance terms in the expression. We set α to 0.2. m was set to 0.2 in the emergency task and 0.3 in the pediatrics task. In *R*_short_, we penalize each hasty stage transition based on the number of features that the actual physician inquired about from the patient in the previous stage but were not inquired about by MedRIA.

Next, we elaborate on the definition of the long-term reward *R*_long_ for the action of terminating the inquiry. When MedRIA selects the action to stop the inquiry process, if the disease predicted by the diagnostic model *D* matches the physician’s diagnosis, *R*_long_ is set to 2 in the emergency task and 3 in the pediatrics task. If it does not match, similar to the penalty of inquiry consistency in *R*_short_, we define *R*_long_(s_t_, x, s_t+1_) as follows: when s_t_ belongs to S_SMH_, *R*_long_(s_t_, x, s_t+1_)=-m·(M_SMH_(s_t_)+M_PE_(s_t_)+M_AE_(s_t_)); when s_t_ belongs to S_PE_, *R*_long_(s_t_, x, s_t+1_)=-m·(M_PE_(s_t_)+M_AE_(s_t_)); when s_t_ belongs to S_AE_, *R*_long_(s_t_, x, s_t+1_)=-m·M_AE_(s_t_).

### Training Details

During the training process of MedRIA, we first trained a VAE. In particular, we simulated the arbitrary partial observations during the inquiry process by randomly dropping a portion of input features. Next, we trained a diagnostic prediction model *D* based on a 5-layer MLP using the softmax function and cross-entropy loss function. Then, we initialized the parameters of the nested VAE in the actor network of MedRIA with the parameters of the trained VAE. Only the nested VAE decoder was fine-tuned in the follow-up training. We used the proximal policy optimization [32] algorithm to train both the actor and critic networks. To make the predicted diagnostic probability distribution more accurate when dealing with the partially observed features generated by MedRIA, we collected the observed features x_O_ when the actor chose to terminate the inquiry process during each training epoch. At the end of each epoch, collected data were used to fine-tune *D* to better adapt to MedRIA’s inquiry patterns. More details on implementation are presented in Multimedia Appendix 3.

### External Validation

We used data from the medical dialogue corpus, IMCS-21 [41], to assess MedRIA’s inquiry logic. IMCS-21 contains 4116 online medical consultations between physicians and patients covering 10 pediatric diseases. After data filtering, we selected 950 consultations with clear diagnoses that overlapped with 4 diseases covered in our pediatrics task: bronchitis (286 consultations), acute upper respiratory tract infection (392 consultations), indigestion (233 consultations), and bronchopneumonia (39 consultations). This data set served as an external test set for the trained pediatric MedRIA. We constructed ordered inquiry sequences based on the dialogue contents. These inquiry sequences reflected the typical order of feature queries in the diagnostic process, and they were used to evaluate MedRIA’s inquiry sequences. If MedRIA’s inquiry sequences are similar to those of physicians, we can conclude that MedRIA’s inquiry logic is similar to that of physicians. The similarity is measured by the Kendall τ distance, which treats an inquiry sequence as a permutation of features and quantifies the pairwise disagreements between 2 permutations. In addition, we applied the normalized Kendall τ distance, which scales the Kendall τ distance by the total number of possible feature pairs.

### Statistical Analysis

Nonparametric bootstrap sampling was used to calculate a 95% CI. Specifically, we repeatedly drew 1000 bootstrap samples from the test set. Each bootstrap sample was obtained through random sampling with replacement and has the same size as the test set.

### Ethical Considerations

All EHR data used in this study were retrospectively collected from EHR systems sourced from routine clinical practice. To ensure patient privacy and confidentiality, all data were deidentified. This study was approved by the ethics committees of the Shenzhen Center for Disease Control and Prevention (SZCDC-IRB2024055). As this study involved no direct patient intervention and was retrospective in nature, individual informed consent was waived.

## Results

### Data Characteristics

We focused on 2 retrospective inquiry-and-diagnosis evaluation tasks in the emergency and pediatrics departments. The emergency departments provided 339,020 records, and the pediatrics departments provided 561,659 records. We randomly split the data into 3 parts: 75% for training MedRIA, 10% for validation, and 15% for testing.

The emergency EHRs contained 862 features, including 42.8% (369/862) SMH features, 21.2% (183/862) PE features, and 36% (310/862) AE features. There were 18 diagnosed diseases. As some records had 2 diagnosed diseases, we classified each combination as a separate disease category, bringing the total number of distinct diagnoses to 23. Similarly, the pediatrics EHRs covered 793 features, including 44.3% (351/793) SMH features, 22.6% (179/793) PE features, and 33.2% (263/793) AE features. There were 18 diagnosed diseases. When combinations of diagnosed diseases were considered, the total number of different diagnoses increased to 23. The detailed data characteristics are presented in Tables 1 and 2.

**Table 1:** Characteristics of the data in the emergency task (N=339,020).

Characteristics		Values
**Gender, n (%)**		
	Male	187,992 (55.5)
	Female	151,028 (44.5)
Age (years), median (IQR)		5.2 (2.6-26.1)
**Diagnosis, n (%)**		
	AURTI^a^	93,594 (27.6)
	Bronchitis	93,564 (27.6)
	Bronchitis with rhinitis	2439 (0.7)
	AURTI with bronchitis	4923 (1.5)
	Bronchopneumonia	3833 (1.1)
	Laryngopharyngitis	6095 (1.8)
	Pharyngitis	24,146 (7.1)
	AURTI with pharyngitis	2639 (0.8)
	Pharyngitis with bronchitis	3564 (1.1)
	Herpangina	7604 (2.2)
	Tonsillitis	10,286 (3)
	AURTI with tonsillitis	1784 (0.5)
	Gastroenteritis	30,385 (9)
	Gastritis	10,142 (3)
	Enteritis	2690 (0.8)
	Acute appendicitis	1957 (0.6)
	Urinary tract stones	13,884 (4.1)
	Urethritis	4052 (1.2)
	Dermatitis	6239 (1.8)
	Acute urticaria	5597 (1.7)
	Hand-foot-and-mouth disease	2990 (0.9)
	Hypertension	4397 (1.3)
	Lumbar disc herniation	2216 (0.7)

^a^AURTI: acute upper respiratory tract infection.

**Table 2:** Characteristics of the data in the pediatrics task (N=561,659).

Characteristics		Values
**Gender, n (%)**		
	Male	315,074 (56.1)
	Female	246,585 (43.9)
Age (years), median (IQR)		3.8 (2.0-5.7)
**Diagnosis, n (%)**		
	Bronchitis	209,918 (37.4)
	AURTI^a^	155,181 (27.6)
	AURTI with bronchitis	11,734 (2.1)
	Bronchopneumonia	8119 (1.4)
	Bronchitis with bronchopneumonia	2565 (0.5)
	Pharyngitis	41,205 (7.3)
	Pharyngitis with bronchitis	5232 (0.9)
	AURTI with pharyngitis	2859 (0.5)
	Laryngopharyngitis	11,909 (2.1)
	Tonsillitis	19,790 (3.5)
	Herpangina	15,647 (2.8)
	Rhinitis	14,824 (2.6)
	Bronchitis with rhinitis	10,109 (1.8)
	Gastroenteritis	16,261 (2.9)
	Gastritis	5313 (0.9)
	Enteritis	3479 (0.6)
	Indigestion	8757 (1.6)
	Hand-foot-and-mouth disease	5156 (0.9)
	Dermatitis	3151 (0.6)
	Acute urticaria	2595 (0.5)
	Developmental delay	2953 (0.5)
	Hyperactivity	2538 (0.5)
	Precocious puberty	2364 (0.4)

^a^AURTI: acute upper respiratory tract infection.

### Evaluation Criteria

We assessed the inquiry performance of MedRIA along 2 dimensions: the consistency of its inquiries with those of physicians and the contribution of its inquiries to diagnostic accuracy. It should be noted that physicians will inevitably make incorrect diagnoses, conduct inquiries based on individual previous experience, and record the EHRs in different ways. Therefore, we can establish a baseline diagnostic model by training a supervised classification model using the extracted feature sets as input and the physicians’ diagnoses as output. This model simulated the physicians’ diagnostic ability, and its performance approximates the actual performance of physicians. We aimed to mitigate the influence of individual physician variability and ensure a fair comparison between different inquiry methods based on the extracted feature sets. While the diagnoses in the records could serve as a gold standard, our simulated physician diagnostic model also serves as an important baseline. For training, several machine learning models were used, including logistic regression, random forest, support vector machine, MLP, and light gradient-boosting machine [42]. We chose light gradient-boosting machine for the emergency task and random forest for the pediatrics task based on their performance on the validation set.

**Figure 2:**
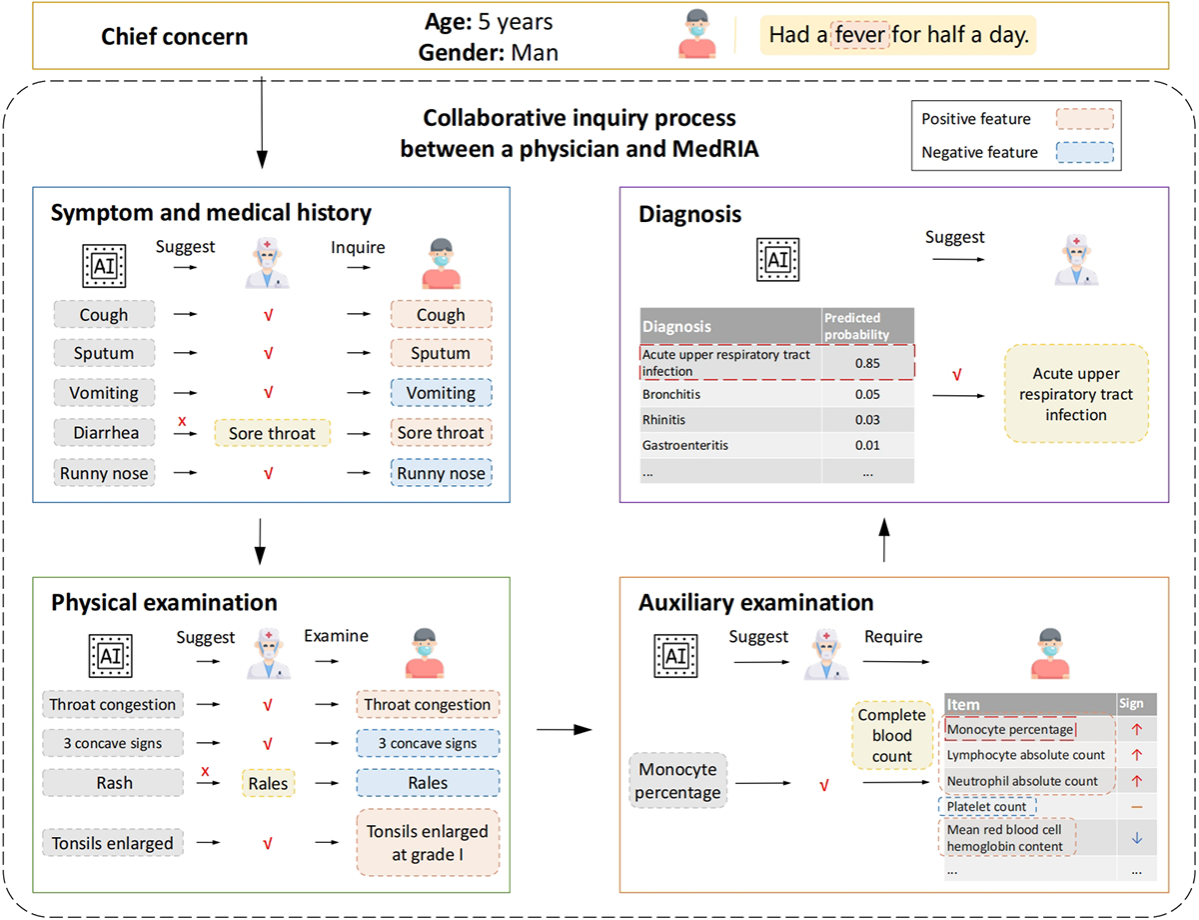
Schematic illustration of the simulated collaborative inquiry process between a physician and MedRIA. AI: artificial intelligence.

To better evaluate the potential of MedRIA as a clinical assistant, we simulated a collaborative inquiry process between MedRIA and a physician. Specifically, MedRIA’s actor network would generate probability distributions for potential inquiry features based on the currently collected medical information. These distributions suggest several high-probability features for the physician to inquire about. The physician has the flexibility to accept and incorporate some or all of these suggestions into the inquiries. In addition, the physician can exercise their professional judgment to either ask additional questions or proceed to examination. In our simulated collaboration, we simplified this process by having MedRIA recommend only the feature with the highest probability at each step. If the inquiry features suggested by MedRIA were in agreement with part of the extracted feature set, it was assumed that the physician accepted the inquiry recommendations. Otherwise, if the suggested features were not found in the extracted feature set, the simulated physician would randomly select an unasked feature from the extracted features as the next inquiry question. This simplification represents a simplistic approximation of a less informed or junior physician’s behavior. It serves as a conservative estimate of MedRIA’s performance when collaborating with less experienced counterparts, reflecting a lower-bound performance of MedRIA-physician collaborative inquiry. Figure 2 illustrates a vivid example of the simulated collaborative inquiry process.

### Inquiry Quality Analysis

Table 3 illustrates the performance of MedRIA in the emergency and pediatrics tasks in terms of diagnosis accuracy and inquiry consistency. In the emergency task, physicians used an average of 13.26 (95% CI 13.202-13.317) inquiries to gather 19.488 (95% CI 19.361-19.618) features. This disparity in numbers is because a single inquiry related to a specific laboratory test can yield multiple features. For example, a complete blood count includes several features, such as lymphocyte percentage, platelet count, hemoglobin concentration, and so on. When MedRIA conducted its own inquiries, it averaged 14.24 (95% CI 14.2-14.284) inquiries, with 58% (8.253/14.24; 95% CI 8.212-8.297) of them matching the extracted features. It resulted in the acquisition of an average of 10.628 (95% CI 10.549-10.714) features, accounting for 55% (10.628/19.488) of all extracted features. In the pediatrics task, MedRIA performed, on average, 1.9 more inquiries (16.271, 95% CI 16.231-16.307) than physicians (14.364, 95% CI 14.322-14.404), with 60% (9.81/16.271; 95% CI 9.776-9.842) of them matching 64% (13.168/20.429; 95% CI 13.093-13.244) of all extracted features (20.429, 95% CI 20.338-20.513). When MedRIA collaborated with physicians, it recalled 95% (18.543/19.488; 95% CI 18.425-18.657) and 99% (20.168/20.429; 95% CI 20.080-20.251) of the extracted features in the emergency and pediatrics tasks, respectively. The simulated collaborative inquiry process required only an average of 12.648 (95% CI 12.602-12.696) and 14.146 (95% CI 14.106-14.182) inquiries, respectively, for the emergency and pediatrics tasks, both fewer than the number of inquiries made by physicians. With the assistance of MedRIA, we were able to reduce the number of inquiries made by physicians to 46% (6.037/13.26; 95% CI 6.009-6.064) and 43% (6.245/14.364; 95% CI 6.225-6.269) of those in independent inquiries while recovering nearly all the original inquiry records.

**Figure 3:**
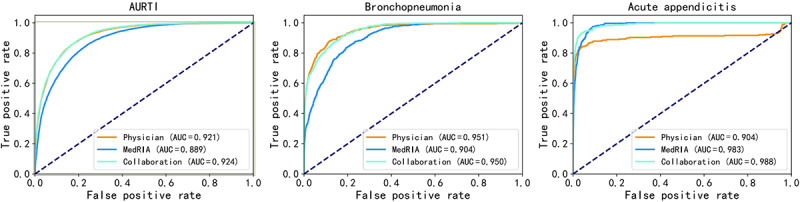
Receiver operating characteristic curves for 3 diseases in the emergency task. AUC: area under the curve; AURTI: acute upper respiratory tract infection.

We used multiple metrics to assess final diagnoses, including the area under the receiver operating characteristic curve (AUROC), macro–*F*_1_-score, and accuracy. We also introduced a new metric called general accuracy, which considers both fully and partially correct diagnoses in accuracy calculation because health care professionals from different hospitals may have varying recording habits. A diagnosis was considered fully correct when the core diagnosis was recorded and partially correct when only a constituent element of the core diagnosis was recorded [43,44]. As shown in Table 3, when MedRIA conducted its own inquiries in the emergency task, it achieved an AUROC (0.955, 95% CI 0.953-0.956) comparable to when it acquired complete extracted features (0.960, 95% CI 0.957-0.962) despite obtaining only 55% (10.628/19.488) of the extracted features. In the pediatrics task, MedRIA achieved a higher AUROC (0.943, 95% CI 0.941-0.944) with 64% (13.168/20.429) of the extracted features compared to using complete features (0.930, 95% CI 0.928-0.932). As shown in Figure 3, MedRIA’s own inquiry and the collaborative inquiry in the emergency task significantly improved the AUROC of diagnostic predictions for acute appendicitis. Specifically, when compared to physicians’ inquiry, both MedRIA’s own inquiry and the collaborative inquiry demonstrated substantially lower false negative rates (equal to 1 minus true positive rates) at various false positive rate thresholds. These findings underscore the potential of MedRIA to significantly reduce diagnostic errors, particularly for critical conditions such as acute appendicitis in which even small increases in false negative rates may be unacceptable to patients and health system leaders.

**Figure 4:**
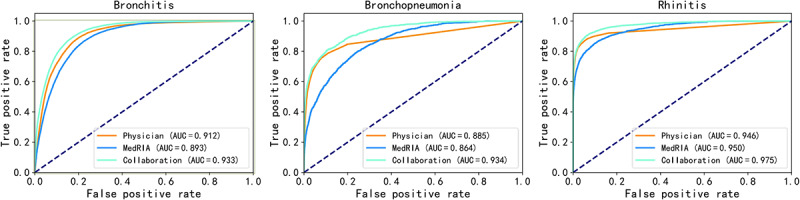
Receiver operating characteristic curves for 3 diseases in the pediatrics task. AUC: area under the curve.

The collaborative inquiry and the physicians’ inquiry resulted in similar AUROCs for acute upper respiratory tract infections and bronchopneumonia. As shown in Figure 4, MedRIA’s own inquiry resulted in a higher AUROC for rhinitis than the physicians’ inquiry in the pediatrics task but a lower AUROC for bronchitis and bronchopneumonia. For these 3 diseases, the collaborative inquiry yielded the highest AUROC. The complete receiver operating characteristic curves for all diseases are shown in Figures S1 and S2 in Multimedia Appendix 4. These results demonstrate that MedRIA understands the importance of inquiring about discriminative features that have a high diagnostic contribution, especially in the pediatrics task.

**Table 3:** Performance of MedRIA in the emergency and pediatrics tasks.

			AUROC^a^ (95% CI)		*F*_1_-score (95% CI)		General accuracy (95% CI)		Accuracy (95% CI)		Average number of inquiries (95% CI)		Average number of inquiries matching extracted features (95% CI)		Average number of inquiries conducted by physicians (95% CI)		Average number of recalled features (95% CI)
**Emergency**																	
	Physicians	0.96 (0.957-0.962)		0.63 (0.623-0.638)		0.853 (0.85-0.856)		0.744 (0.741-0.748)		13.26 (13.202-13.317)		13.26 (13.202-13.317)		13.26 (13.202-13.317)		19.488 (19.361-19.618)	
	MedRIA	0.955 (0.953-0.956)		0.538 (0.531-0.545)		0.806 (0.803-0.81)		0.684 (0.68-0.688)		14.24 (14.2-14.284)		8.253 (8.212-8.297)		0		10.628 (10.549-10.714)	
	Collaboration	0.972 (0.97-0.973)		0.646 (0.639-0.653)		0.854 (0.851-0.857)		0.746 (0.742-0.75)		12.648 (12.602-12.696)		12.648 (12.602-12.696)		6.037 (6.009-6.064)		18.543 (18.425-18.657)	
**Pediatrics**																	
	Physicians	0.93 (0.928-0.932)		0.613 (0.608-0.618)		0.827 (0.825-0.83)		0.704 (0.701-0.708)		14.364 (14.322-14.404)		14.364 (14.322-14.404)		14.364 (14.322-14.404)		20.429 (20.338-20.513)	
	MedRIA	0.943 (0.941-0.944)		0.521 (0.514-0.527)		0.782 (0.779-0.784)		0.656 (0.653-0.66)		16.271 (16.231-16.307)		9.81 (9.776-9.842)		0		13.168 (13.093-13.244)	
	Collaboration	0.968 (0.967-0.969)		0.633 (0.627-0.638)		0.842 (0.839-0.844)		0.732 (0.729-0.735)		14.146 (14.106-14.182)		14.146 (14.106-14.182)		6.245 (6.225-6.269)		20.168 (20.08-20.251)	

^a^AUROC: area under the receiver operating characteristic curve.

MedRIA may conduct inquiries that were not asked by physicians, so they are excluded from the extracted features of patient EHRs. Hence, when MedRIA conducted its own inquiries, there was a varying degree of decline in all diagnostic metrics when compared to using complete extracted features. On the other hand, MedRIA obtained more information through the guidance of physicians when it collaborated with them. In the emergency task, the *F*_1_-score (0.646, 95% CI 0.639-0.653) of diagnoses significantly improved. Both general accuracy (0.854, 95% CI 0.851-0.857) and accuracy (0.746, 95% CI 0.742-0.750) were comparable to those of physicians’ diagnoses. In the pediatrics task, collaborative inquiry led to a significant increase in *F*_1_-score (0.633, 95% CI 0.627-0.638), general accuracy (0.842, 95% CI 0.839-0.844), and accuracy (0.732, 95% CI 0.729-0.735).

Considering the high prevalence of respiratory diseases in our data set, we evaluated MedRIA’s performance on a more representative data set to show its generalizability. Specifically, we randomly discarded two-thirds of respiratory cases from the emergency data set and three-quarters from the pediatrics data set. The results are presented in Table S1 in Multimedia Appendix 5. From these results, we can draw similar conclusions to those obtained from the experiments conducted on the complete data set.

### Inquiry Logic Analysis

Table 4 provides the number of different types of positive recalled features, which refer to exhibited symptoms or laboratory test results showing out-of-range values. Collecting more positive features under the same number of inquiries often leads to more accurate diagnosis results [16,45]. In the pediatrics task, 36% (7.326/20.429; 95% CI 7.294-7.357) of the features collected by physicians were positive, and MedRIA’s own inquiries recalled 66% (4.852/7.326; 95% CI 4.825-4.877) of positive features. Specifically, it recalled 62% (1.665/2.692; 95% CI 1.654-1.675) of SMH features, 79% (2.24/2.837; 95% CI 2.23-2.25) of PE features, and 53% (0.948/1.797; 95% CI 0.931-0.965) of AE features. When MedRIA worked with physicians, the recall rates for all types of features were >95%. Overall, we observed that MedRIA was capable of accurately identifying positive features that were not mentioned in the chief concerns of patients. This ability is crucial during the investigation process because patients may not always have sufficient medical knowledge to fully describe their health conditions.

**Figure 5:**
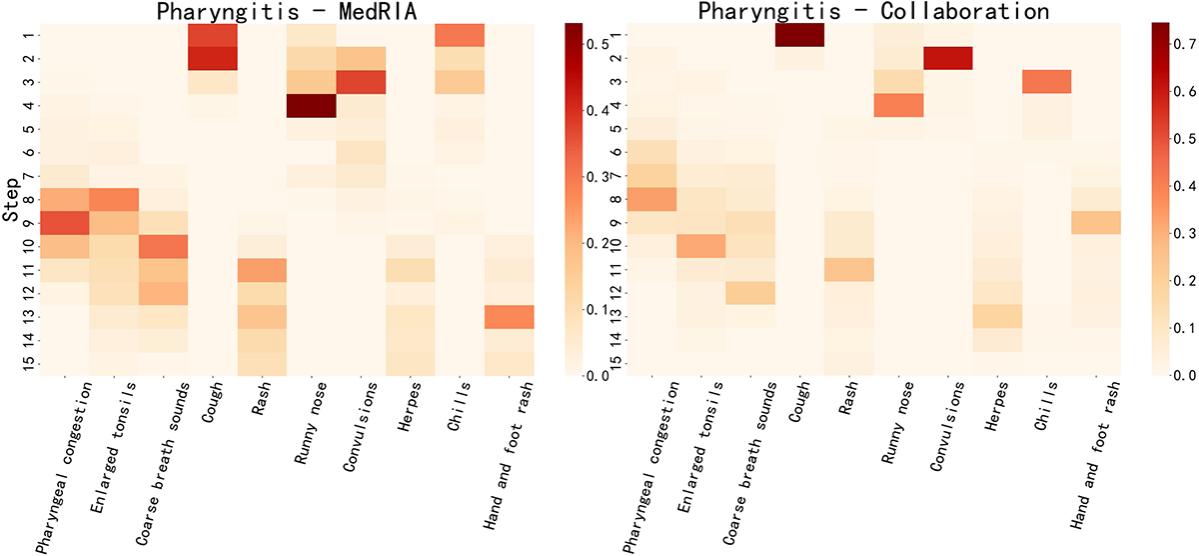
MedRIA and collaborative inquiry heat maps of the top 10 physician inquiry features in patients diagnosed with pharyngitis in the emergency task.

**Figure 6:**
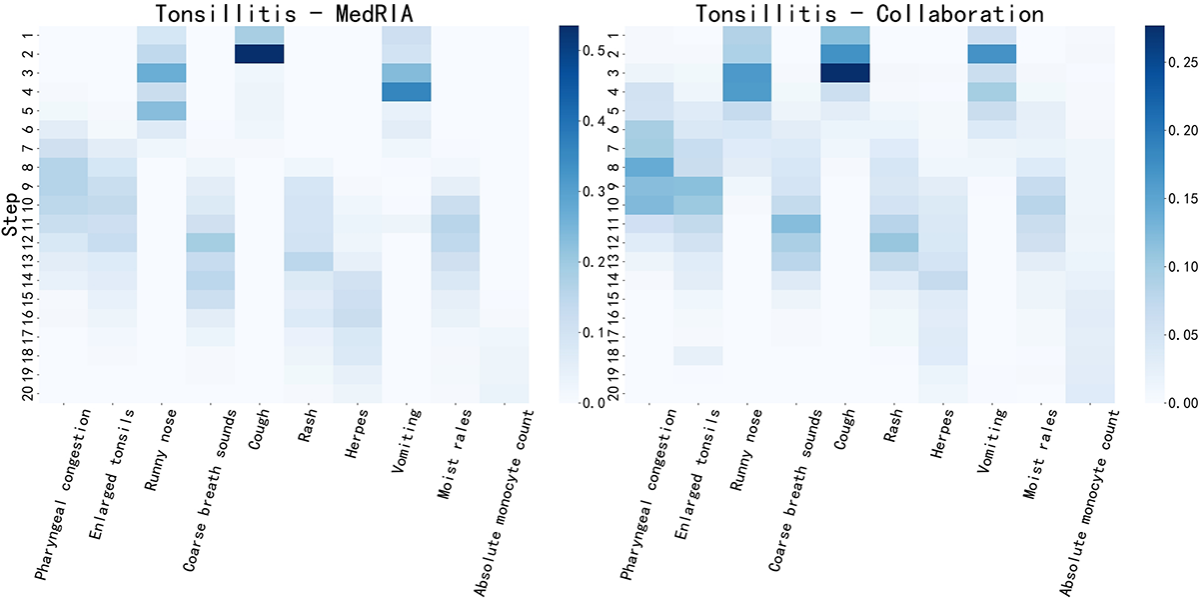
MedRIA and collaborative inquiry heat maps of the top 10 physician inquiry features in patients diagnosed with tonsillitis in the pediatrics task.

Multimedia Appendix 6 shows the top 10 physician inquiry features on patients diagnosed with pharyngitis in the emergency task and tonsillitis in the pediatrics task. We observed that MedRIA could accurately inquire about features obtained by physicians. Figures 5 and 6 illustrate when these features were investigated by MedRIA and through collaborative investigation, respectively. First, we observed a clear distinction between when SMH and PE features were acquired, which is in line with our settings for MedRIA’s inquiry process. SMH features, including cough, runny nose, convulsions, and chills, were consistently acquired before the seventh step, whereas PE features, including pharyngeal congestion, enlarged tonsils, coarse breath sounds, rash, herpes, and hand and foot rash, were consistently acquired after the seventh step. We also noticed that consecutive steps of inquiries exhibited consistently high frequencies, allowing us to understand MedRIA’s inquiry logic clearly. For example, when conducting PEs, MedRIA began with pharyngeal congestion and enlarged tonsils followed by coarse breath sounds and rash. This logical sequence reflects a structured approach to differential diagnosis, ensuring a thorough evaluation of relevant symptoms and signs. Pharyngeal congestion and enlarged tonsils are pivotal features in the context of pharyngitis. Coarse breath sounds may suggest respiratory issues or lung conditions, whereas rash can be associated with a wide range of dermatological, allergic, or infectious disorders. As shown in Figures 5 and 6, when collaborating with physicians, the refinement of MedRIA’s inquiry logic is evident in the increased stability of determining which features to inquire about at each step. Table 5 provides concrete examples of MedRIA’s inquiry sequences, where the aforementioned logic can be traced back. Figure S1 in Multimedia Appendix 7 presents the tree diagram depicting the order of MedRIA’s inquiry features for 4 patients diagnosed with pharyngitis in the emergency task. These evidences demonstrate MedRIA’s coherent, stable, and well-reasoned inquiry logic. The characteristics of inquiry features for all diseases in both tasks are shown in Figures S1-S6 in Multimedia Appendix 8.

**Table 4:** Number of recalled features from the extracted features by MedRIA in the emergency and pediatrics tasks.

		Average SMH^a^ features (95% CI)	Average PE^b^ features (95% CI)	Average AE^c^ features (95% CI)	Average positive features^d^ (95% CI)	Average positive SMH features (95% CI)	Average positive PE features (95% CI)	Average positive AE features (95% CI)
**Emergency**								
	Physicians	7.295 (7.26-7.332)	5.291 (5.265-5.32)	6.902 (6.793-7.011)	6.83 (6.788-6.87)	2.52 (2.503-2.538)	2.527 (2.514-2.541)	1.782 (1.749-1.813)
	MedRIA	4.31 (4.29-4.331)	3.835 (3.808-3.865)	2.483 (2.421-2.546)	3.923 (3.897-3.954)	1.363 (1.353-1.375)	1.907 (1.894-1.92)	0.653 (0.635-0.672)
	Collaboration	7.01 (6.978-7.043)	5.222 (5.196-5.25)	6.311 (6.206-6.415)	6.543 (6.505-6.581)	2.435 (2.418-2.452)	2.501 (2.489-2.515)	1.607 (1.576-1.636)
**Pediatrics**								
	Physicians	7.522 (7.494-7.546)	6.29 (6.269-6.312)	6.617 (6.546-6.687)	7.326 (7.294-7.357)	2.692 (2.677-2.707)	2.837 (2.826-2.848)	1.797 (1.776-1.82)
	MedRIA	5.021 (5.001-5.04)	4.639 (4.619-4.66)	3.508 (3.452-3.565)	4.852 (4.825-4.877)	1.665 (1.654-1.675)	2.24 (2.23-2.25)	0.948 (0.931-0.965)
	Collaboration	7.461 (7.435-7.484)	6.28 (6.258-6.302)	6.427 (6.356-6.496)	7.241 (7.209-7.27)	2.672 (2.658-2.687)	2.833 (2.822-2.844)	1.736 (1.714-1.758)

^a^SMH: symptom and medical history.

^b^PE: physical examination.

^c^AE: auxiliary examination.

^d^Refers to exhibited symptoms or laboratory test results showing out-of-range values.

**Table 5:** Examples of the inquiry sequences of MedRIA on 3 patients diagnosed with pharyngitis.

Gender	Age (y)	Chief concern	Inquiry sequences of SMH^a^	Inquiry sequences of PE^b^	Inquiry sequences of AE^c^
Male	26	*Sore throat* ^d^	*Fever(0)*^e^, *cough(0)*, *runny nose(0)*, *nasal congestion(0)*, *chest pain(0)*, *coughing up phlegm(0)*, abdominal pain, diarrhea	*Pharyngeal congestion(1)*, *enlarged tonsils(0)*, *moist rales(0)*, *dry rales(0)*, lower limb edema, *coarse breath sounds(0)*	*Absolute monocyte count [complete blood count]* ^f^
Male	50	*Pharyngeal foreign body sensation*	Fever, *cough(1)*, *sore throat(0)*, runny nose, *coughing up phlegm(0)*, abdominal pain, diarrhea, vomiting, dizziness	*Pharyngeal congestion(1)*, *enlarged tonsils(0)*, *moist rales(0)*, *dry rales(0)*, *coarse breath sounds(0)*, rash	Absolute monocyte count
Male	4	*Fever*	*Cough(1)*, runny nose, *vomiting(0)*, coughing up phlegm, *diarrhea(0)*, *convulsions(0)*, *shortness of breath(0)*	*Pharyngeal congestion(1)*, *coarse breath sounds(1)*, *enlarged tonsils(0)*, *hand and foot Rash(0)*, *dry rales(0)*, normal muscle tone, *moist rales(0)*	*Absolute monocyte count [complete blood count]*

^a^SMH: symptom and medical history.

^b^PE: physical examination.

^c^AE: auxiliary examination.

^d^Features belonging to the extracted features are italicized.

^e^Feature values are indicated in parentheses.

^f^Contents within square brackets represent the associated laboratory tests documented in the patient’s health record.

### External Validation

We used external medical dialogues to evaluate MedRIA’s inquiry logic. These dialogues consist of inquiry sequences generated by physicians during medical consultations. Limited by the online scenario, the average length of the inquiry sequences was 3.905 (95% CI 3.744-4.076), consisting of 3.043 (95% CI 2.895-3.195) inquiries about SMH and PE features and 0.862 (95% CI 0.814-0.907) recommended AEs. Diagnoses based on such few features were unreliable, so we primarily focused on evaluating MedRIA’s own inquiry order. As a result, MedRIA matched 64% (1.946/3.043; 95% CI 1.835-2.058) of the inquiries in the inquiry sequences and 28% (0.244/0.862; 95% CI 0.218-0.269) of recommended AEs. We compared the order of MedRIA’s inquiries to the inquiry sequences of physicians. The normalized Kendall τ distance was 0.323 (95% CI 0.301-0.346). It indicated that only 32.3% of pairs of inquiry features differed in ordering, demonstrating MedRIA’s consistency with physicians and reasonable inquiry logic.

## Discussion

### Principal Findings

In this study, we proposed a reinforcement learning medical inquiry assistant, MedRIA, aiming to provide inquiry recommendations for medical investigation processes. The variety of questions that can be asked during the medical investigation process, especially when it incorporates PEs and AEs, poses a significant challenge. In addition, patients vary in their understanding of their health status and their ability to articulate it. To address these issues, MedRIA uses an actor-critic framework [28]. The actor incorporates a pretrained VAE [31] to recommend actions based on conditional probability distributions between observed and unobserved patient information. MedRIA uses a supervised diagnostic prediction model to aid in action selection, ensuring that the actor suggests inquiries that contribute to accurate diagnoses. In addition, MedRIA places significant emphasis on crafting a reward function to guide the reinforcement learning process.

The superiority of these designs in MedRIA was verified in our retrospective experiments. When conducting its own inquiries in both emergency and pediatrics scenarios, MedRIA demonstrated comparable diagnostic prediction performance on AUROCs with that of physicians and even achieved a higher AUROC in the pediatrics setting. This was accomplished with MedRIA obtaining only 55% (10.628/19.488) and 64% (13.168/20.429) of the information that physicians received in making the final diagnosis in the emergency and pediatrics tasks, respectively. It is important to consider that AI, similarly to MedRIA, has the capacity to identify subtle patterns within vast data sets, which physicians may overlook. This inherent capability often leads to divergence from the inquiry logic of individual clinicians, as evidenced in our experiments. Given that MedRIA has demonstrated its ability to identify important diagnostic features, such divergence should be considered acceptable as it may provide new insights. In addition, it is worth noting that our experiments were based on EHRs. Therefore, when MedRIA conducted its own inquiries, its performance might very well have been underestimated due to the unavailability of patient information that was not documented in the records.

When MedRIA worked in simulated collaboration with physicians, it notably reduced the number of inquiries made by physicians to 46% (6.037/13.26; 95% CI 6.009-6.064) and 43% (6.245/14.364; 95% CI 6.225-6.269) while maintaining feature recall rates of 95% (18.543/19.488; 95% CI 18.425-18.657) and 99% (20.168/20.429; 95% CI 20.080-20.251) in the emergency and pediatrics scenarios, respectively. This reduction suggests that MedRIA’s recommendations are sufficiently informative to assist physicians in considering aspects they may have overlooked. The additional insights provided by AI recommendations have a significant impact on medical decision-making [46]. By leveraging the insights provided by MedRIA, physicians can potentially make more informed decisions and arrive at more accurate diagnoses.

The demonstration and analysis of MedRIA’s inquiry feature frequencies, as well as concrete inquiry sequences, reveal its clear inquiry logic for different diseases, which resembles the approach of physicians. The ability of MedRIA to emulate the diagnostic process of human physicians suggests that it has the potential to be a useful tool in medical education, particularly in regions or populations with limited medical resources. Junior health care practitioners can leverage MedRIA to enhance their diagnostic skills, benefiting from its structured and logical inquiry process.

### Limitations

Despite these promising results, it is essential to acknowledge certain limitations. First, the feature extraction process of EHRs requires careful feature engineering, which could be further refined. Second, while MedRIA excelled in adapting its inquiry logic, it is not immune to instances of missed inquiries. For example, the reliance on AEs of MedRIA is significantly lower than that of physicians. One potential solution is to enhance MedRIA by modeling the significance of inquiries for disease treatment. This is because the purpose of examinations may not be for diagnosis but to determine the severity of the disease to formulate appropriate treatment.

Third, the composition of patient presentations can vary significantly between countries. For example, in some Western countries, only 5% to 20% of emergency [47] or pediatrics [48] outpatients are due to upper respiratory tract infections. The proportion in our data set was many times greater due to seasonal variations. We speculate that this may have arisen due to limited access to primary care, seasonal respiratory peaks during the autumn and winter months, greater COVID-19 awareness and fear, and our method of prioritizing the most frequent diagnoses. Changes in these factors, such as using summer data in China or winter data elsewhere, may influence accuracy. Furthermore, while MedRIA’s workflow is language independent, the evaluation of our system has been limited to a Chinese context. Further validation in other languages and health care settings is necessary to establish its broader applicability and effectiveness.

Fourth, our AI system would be biased toward excess certainty if deployed in the real world as it lacks the humility to recognize its diagnostic limitations. Future work should include the AI system’s degree of confidence and manage corresponding clinical risks. Fifth, decision system developers must consider adopting safe integration systems to mitigate potential errors, especially for life-threatening conditions such as appendicitis, where risk aversion may be greater. Sixth, future work could incorporate MedRIA with the latest developments in LLMs [49], which will assist in its future deployment and testing in clinical workflows.

### Conclusions

In conclusion, MedRIA is a significant step toward enhancing health care decision-making processes. Its proficiency in medical inquiries, ability to identify important diagnostic features, and adaptability underscore its value as an AI-driven diagnostic assistant. While there are challenges to overcome, the results of this study show a prototype for sophisticated and effective AI applied in health care, ultimately benefiting both health care professionals and patients.

## Appendix

Multimedia Appendix 1 The feature set of the emergency task.

Multimedia Appendix 2 The feature set of the pediatrics task.

Multimedia Appendix 3 Implementation details of MedRIA.

Multimedia Appendix 4 Receiver operating characteristic curves of all diseases in the emergency and pediatrics tasks.

Multimedia Appendix 5 Performance of MedRIA in more generalizable emergency and pediatrics tasks.

Multimedia Appendix 6 Inquiry frequencies of the top 10 physician inquiry features in patients diagnosed with pharyngitis in the emergency task and tonsillitis in the pediatrics task.

Multimedia Appendix 7 Tree diagram of the inquiry features of MedRIA for 4 patients diagnosed with pharyngitis in the emergency task. These 4 patients shared similar characteristics, all being male with ages ranging from 31 to 32 years and complaining of sore throat. The numbers in brackets indicate the timesteps of inquiries.

Multimedia Appendix 8 Inquiry frequencies of the physician and MedRIA and inquiry heat maps of MedRIA and the collaborative inquiry in the emergency and pediatrics tasks.

## Abbreviations

AE: auxiliary examination
AI: artificial intelligence
AUROC: area under the receiver operating characteristic curve
CDSS: clinical decision support system
EHR: electronic health record
LLM: large language model
MedBERT: Medical Bidirectional Encoder Representations From Transformers
MLP: multilayer perceptron
PE: physical examination
SMH: symptom and medical history
VAE: variational autoencoder
