# Supplementary material for: AI-Driven Diagnostic Assistance in Medical Inquiry: Reinforcement Learning Algorithm Development and Validation
Source: J Med Internet Res. 2024 Aug 23;26:e54616. doi: 10.2196/54616 (PMC11380057; doi:10.2196/54616)
Supplement: Multimedia Appendix 3 [file jmir_v26i1e54616_app3.docx]

**Implementation Details**

**Data De-identification Process**

The de-identification process of collected electronic health records (EHRs) involved several steps to ensure patient privacy and confidentiality. Firstly, all personally identifiable information, such as patient names, ID card numbers, addresses, social security numbers, and contact details, were removed from the EHRs. Secondly, any identifying information related to healthcare providers, such as names and identifiers, was also de-identified to prevent potential re-identification. Finally, health record numbers were replaced with unique, randomly generated identifiers to further anonymize the data.

**Date Cleaning and Filtering**

At first, we removed records from which no features could be extracted. Next, our study exclusively focused on patients of initial consultations, as follow-up visits typically prioritize observation or treatment rather than diagnostic inquiries. Consequently, we excluded records with insufficient diagnostic complexity, wherein patients had already declared their disorders that they were suffering from in chief complaints or history of past illness. Records without definitive disease diagnoses, such as those with “cough” or “diarrhea” in the diagnosis field, were also excluded. Furthermore, we removed all COVID-19 screening results. This decision was influenced by the context of the COVID-19 pandemic in 2021, where individuals with flu-like symptoms in China were mandated to undergo COVID-19 screenings. Since patients testing positive for COVID-19 were either immediately isolated or hospitalized, the screening results in outpatient medical records, all of which were negative, had no bearing on the diagnosis. For the emergency dataset, we excluded data from the emergency surgery department. Patients visiting the emergency surgery department often prioritize immediate treatment, with diagnosis being of secondary importance.

**Training Details**

During the training process of MedRIA, we first used disease diagnoses together with the extracted features to train a variational autoencoder (VAE) [1]. The disease diagnosis was treated as an additional feature and served to help model the conditional probability distribution between features during training. The VAE is trained by optimizing the Evidence Lower Bound (ELBO): $\mathbb{E}_{q_{\Phi}(\mathbf{z}|\mathbf{x})}[\log p_{\Theta}(\mathbf{x}|\mathbf{z})]-D_{\text{KL}}[q_{\Phi}(\mathbf{z}|\mathbf{x})\parallel p(\mathbf{z})]$, where $D_{\text{KL}}$ is the Kullback-Leibler (KL) divergence between two distributions. Utilizing stochastic gradient descent and the reparameterization trick [1] to optimize the ELBO, the VAE was trained to recover the input features by decoding the encoded latent variable. In particular, we simulated the arbitrary partial observations during the inquiry process by randomly dropping a portion of input features before they were fed into the encoder of VAE. We stopped the training of the VAE model when the ELBO on the validation set showed signs of convergence, indicating that the model had sufficiently learned the underlying data distribution. Next, we trained a diagnostic prediction model $\mathcal{D}$ based on a five-layer multilayer perceptron (MLP) using the Softmax function and cross-entropy loss function. During training of the MLP, the input is the complete extracted features $\mathbf{x}$ concatenated with the latent variable obtained from the VAE's encoding of the features $\mathbf{x}$. The concatenation aims to leverage the capabilities of encoding partially observed features of the generative model to enhance the performance of the classification model.

Then, we initialized the parameters of the nested VAE in the actor network of MedRIA with the parameters of the trained VAE. We used the Proximal Policy Optimization (PPO) [2] algorithm to train both the actor and critic networks. Specifically, the critic network was trained using the mean squared error loss function $L_{V}=\frac{1}{n}\sum_{i=1}^{n} (V_{pred}-V_{\pi}(\mathbf{s}_{t}))^{2}$, where $V_{pred}$ is the predicted state-value of the critic network. Following the actor-critic algorithm [3] and utilizing the Generalized Advantage Estimator (GAE) [4], the advantage function optimized at timestep $t$ is ${\overset{^}{A}}_{t}=\sum_{l=0}^{T} (\gamma\lambda)^{l}\delta_{t+l}^{V}$, where $\delta_{t}^{V}=r_{t}+\gamma V(\mathbf{s}_{t+1})-V(\mathbf{s}_{t})$. $T$ is the timestep when the inquiry process terminates, $\gamma$ is a hyperparameter of discount factor, and $\lambda$ is a hyperparameter of GAE. Let $\theta$ be the parameters of the actor network, $o_{t}(\theta)=\frac{\pi_{\theta}(a_{t}|\mathbf{s}_{t})}{\pi_{\theta_{old}}(a_{t}|\mathbf{s}_{t})}$ represents the probability ratio between old and updated policies and $E(\theta)=-\pi_{\theta}(a_{t}|\mathbf{s}_{t})ln(\pi_{\theta}(a_{t}|\mathbf{s}_{t}))$ is the entropy of the policy. Then the actor network was trained to maximize the clipped objective function: $L(\theta)={\overset{^}{\mathbb{E}}}_{t}[min(o_{t}(\theta){\overset{^}{A}}_{t},clip(o_{t}(\theta),1-\epsilon,1+\epsilon){\overset{^}{A}}_{t})+\eta\cdot E(\theta)]$, where $\eta$ is a small hyperparameter to control the entropy of the policy to encourage or restrain exploration. To avoid repeatedly inquiring about the same features during training, we set the probabilities of actions that have already been selected by the actor in the action probability distribution to zero.

The diagnostic probability distribution from the diagnostic prediction model $\mathcal{D}$ was used in both actor and critic during training. To make the predicted diagnostic probability distribution more accurate when dealing with the partially observed features during the inquiry process, we collected the observed features $\mathbf{x}_{O}$ when the actor chose to terminate the inquiry process during each training epoch. At the end of the epoch, this data was used to fine-tune the diagnostic prediction model to better adapt to MedRIA's inquiry patterns.

We trained all models using the Adam optimizer [5] with the batch size set to 512. The learning rates of both the VAE model and the diagnostic prediction model $\mathcal{D}$ were $1\times{10}^{-4}$, and $5\times{10}^{-5}$ for both the actor and critic networks. The learning rates would linearly decay to one-tenth with iterations. In the actor’s objective function $L(\theta)$, we set η to 0.01. The discount factor $\gamma$ for accumulating rewards at each step was set to 0.95. We trained the actor and critic for 500 epochs and started fine-tuning the diagnostic prediction model $\mathcal{D}$ at the 63th epoch. Each epoch contains 409,600 and 512,000 inquiry steps for the emergency and pediatrics task, respectively. MedRIA was implemented using PyTorch [6] and Tianshou [7].

The upper limit of action count $N_{T}$ was set to 21 and 26 for the emergency and pediatrics task, respectively. In the simulated collaborative inquiry process, if MedRIA chose to inquire about a feature that was not in extracted features, we randomly selected an unasked feature as the suggested inquiry question by the physician. We first performed selection from the unasked SMH features. If there were no unasked SMH features, we would proceed to the unasked PE features, and then unasked AE features.

## References

1. Kingma DP, Welling M. Auto-encoding variational Bayes. Preprint at <https://arxiv.org/abs/1312.6114>. 2013.

2. Schulman J, Wolski F, Dhariwal P, Radford A, Klimov O. Proximal policy optimization algorithms. Preprint at <https://arxiv.org/abs/1707.06347>. 2017.

3. Konda V, Tsitsiklis J. Actor-critic algorithms. Advances in Neural Information Processing Systems: volume 12. 1999.

4. Schulman J, Moritz P, Levine S, Jordan M, Abbeel P. High-dimensional continuous control using generalized advantage estimation. Preprint at <https://arxiv.org/abs/1506.02438>. 2015.

5. Kingma DP, Ba J. Adam: A method for stochastic optimization. Preprint at <https://arxiv.org/abs/1412.6980>. 2014.

6. Paszke A, Gross S, Massa F, Lerer A, Bradbury J, Chanan G, et al. PyTorch: An imperative style, high-performance deep learning library. Advances in Neural Information Processing Systems: volume 32. 2019.

7. Weng J, Chen H, Yan D, You K, Duburcq A, Zhang M, et al. Tianshou: A highly modularized deep reinforcement learning library. Journal of Machine Learning Research. 2022;23(267):1-6.
