# Supplementary material for: AI-Driven Diagnostic Assistance in Medical Inquiry: Reinforcement Learning Algorithm Development and Validation
Source: J Med Internet Res. 2024 Aug 23;26:e54616. doi: 10.2196/54616 (PMC11380057; doi:10.2196/54616)

# **Figure S1: Receiver operating characteristic curves of different diseases in the emergency task.**


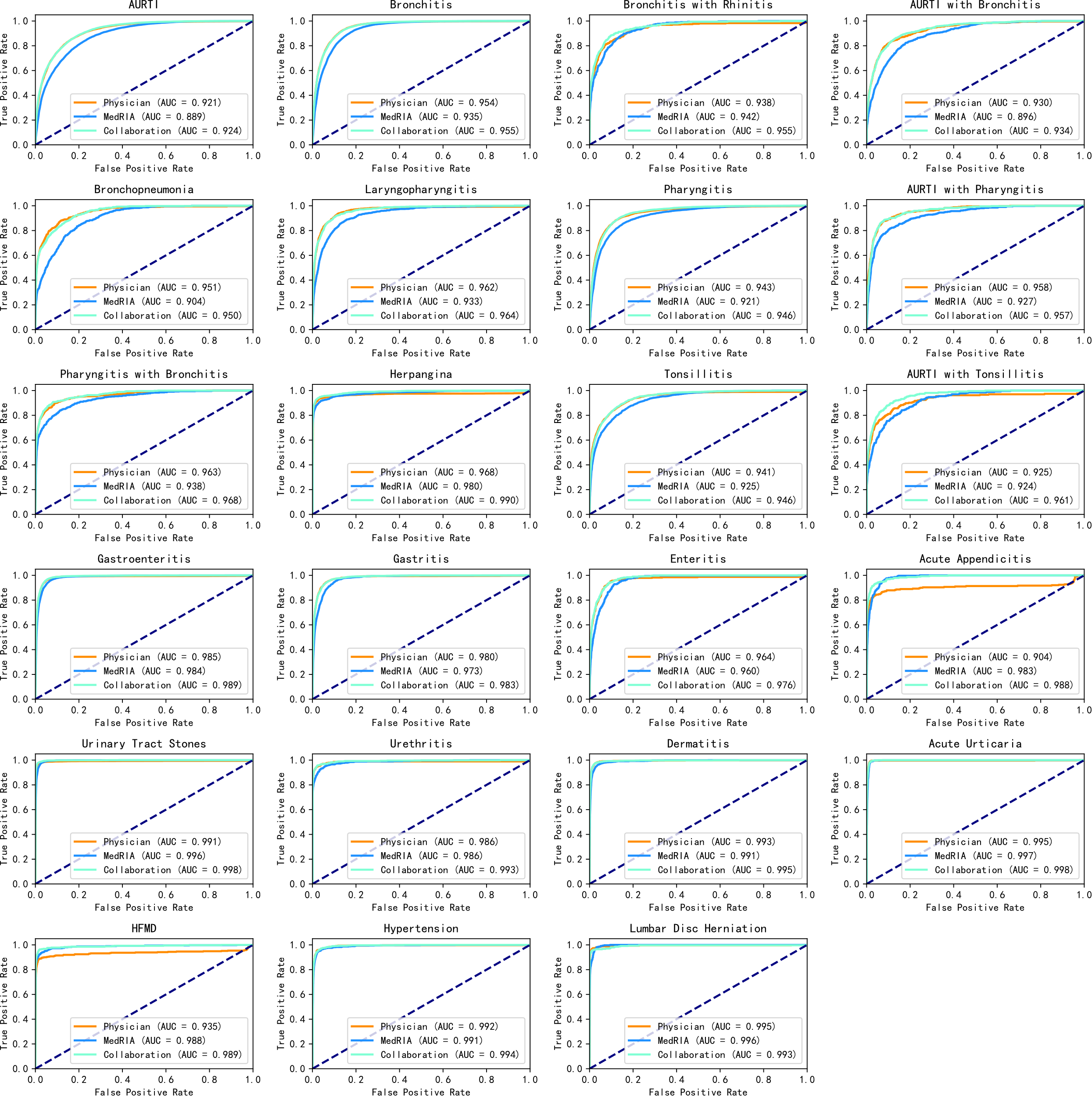


# **Figure S2: Receiver operating characteristic curves of different diseases in the pediatrics task.**


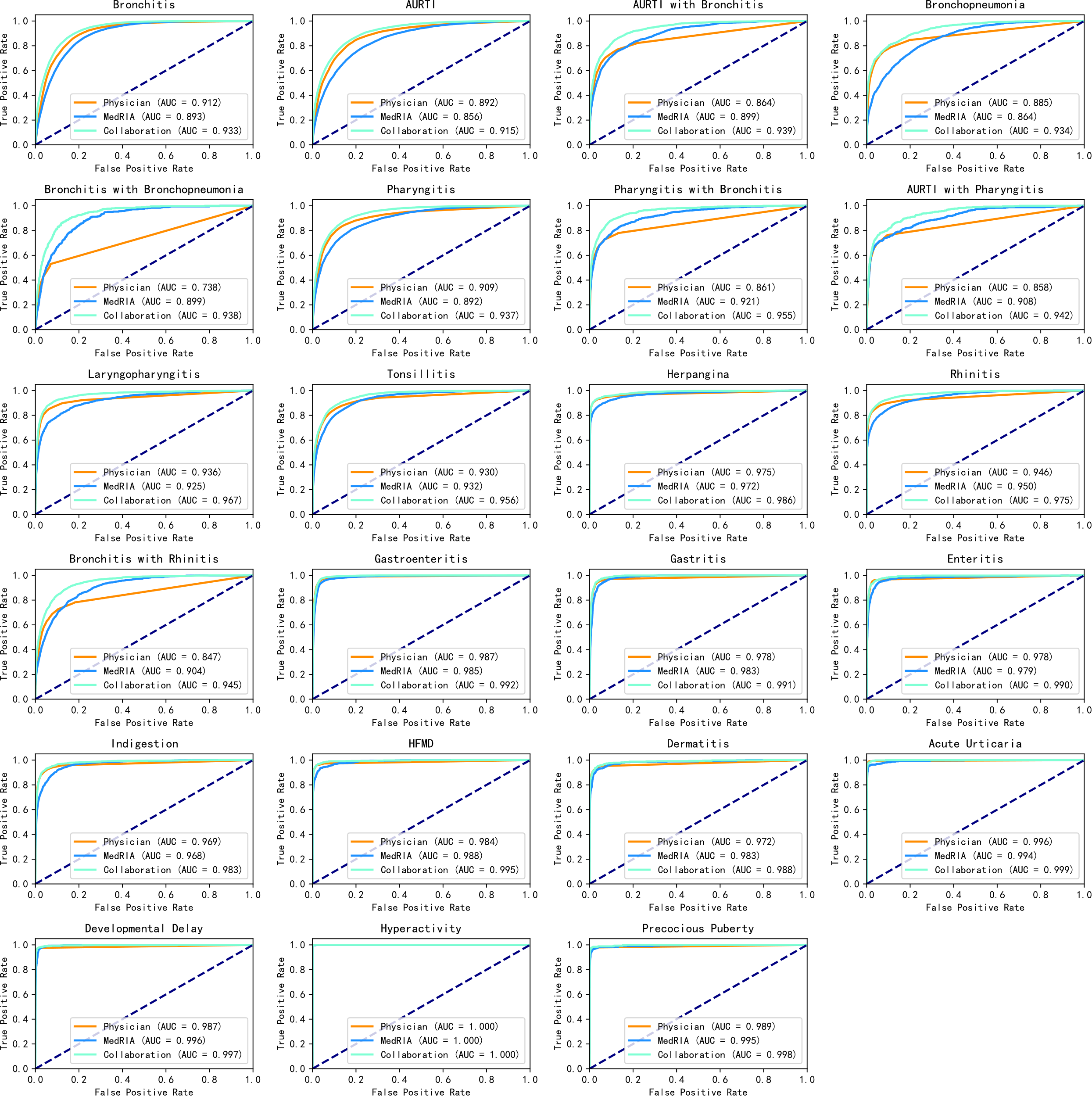

Supplement: Multimedia Appendix 4 [file jmir_v26i1e54616_app4.docx]
