# Supplementary material for: AI-Driven Diagnostic Assistance in Medical Inquiry: Reinforcement Learning Algorithm Development and Validation
Source: J Med Internet Res. 2024 Aug 23;26:e54616. doi: 10.2196/54616 (PMC11380057; doi:10.2196/54616)
Supplement: Multimedia Appendix 5 [file jmir_v26i1e54616_app5.docx]

Table S1. Performance of MedRIA in more generalizable emergency and pediatrics tasks.^a^

|  | AUROC | *F*_1_-score | Gen. acc.^b^ | Acc.^c^ | Inqs.^d^ | Matching inqs.^e^ | Inqs. by phys.^f^ | Recalled features |
| --- | --- | --- | --- | --- | --- | --- | --- | --- |
|  |  |  |  |  |  |  |  |  |
| **Emergency** ^g^ |  |  |  |  |  |  |  |  |
| Physicians | 0.97  (0.968, 0.973) | 0.639  (0.629, 0.649) | 0.867  (0.863, 0.871) | 0.771  (0.766, 0.776) | 12.674  (12.588, 12.759) | 12.674  (12.588, 12.759) | 12.674  (12.588, 12.759) | 19.795  (19.617, 19.966) |
| MedRIA | 0.959  (0.957, 0.961) | 0.524  (0.514, 0.534) | 0.807  (0.802, 0.812) | 0.69  (0.684, 0.696) | 14.1  (14.041, 14.157) | 6.596  (6.548, 6.643) | 0 | 9.484  (9.37, 9.609) |
| Collaboration | 0.971  (0.97, 0.973) | 0.628  (0.617, 0.638) | 0.861  (0.856, 0.865) | 0.759  (0.755, 0.765) | 11.986  (11.919, 12.051) | 11.986  (11.919, 12.051) | 6.083  (6.046, 6.118) | 18.828  (18.647, 18.993) |
| **Pediatrics** ^h^ |  |  |  |  |  |  |  |  |
| Physicians | 0.93  (0.926, 0.934) | 0.602  (0.593, 0.609) | 0.821  (0.816, 0.825) | 0.707  (0.701, 0.712) | 13.812  (13.743, 13.889) | 13.812  (13.743, 13.889) | 13.812  (13.743, 13.889) | 19.48  (19.323, 19.638) |
| MedRIA | 0.925  (0.921, 0.928) | 0.505  (0.495, 0.512) | 0.755  (0.75, 0.759) | 0.632  (0.626, 0.638) | 15.909  (15.841, 15.979) | 8.676  (8.628, 8.731) | 0 | 12.162  (12.035, 12.287) |
| Collaboration | 0.96  (0.958, 0.963) | 0.624  (0.614, 0.633) | 0.827  (0.822, 0.831) | 0.724  (0.718, 0.729) | 13.617  (13.553, 13.688) | 13.617  (13.553, 13.688) | 6.443  (6.405, 6.481) | 19.269  (19.112, 19.422) |

^a^All evaluation metrics are with 95% confidence intervals in brackets.

^b^General accuracy.

^c^Accuracy.

^d^Number of inquiries.

^e^Number of inquiries matching extracted features.

^f^Number of inquiries conducted by physicians.

^g^We randomly discarded two-thirds of respiratory cases in original emergency cohort, resulting in 169,364 records.

^h^ We randomly discarded three-quarters of respiratory cases in original pediatrics cohort, resulting in 179,832 records.
