# Supplementary figures and images for: AI-Driven Diagnostic Assistance in Medical Inquiry: Reinforcement Learning Algorithm Development and Validation
Source: J Med Internet Res. 2024 Aug 23;26:e54616. doi: 10.2196/54616 (PMC11380057; doi:10.2196/54616)

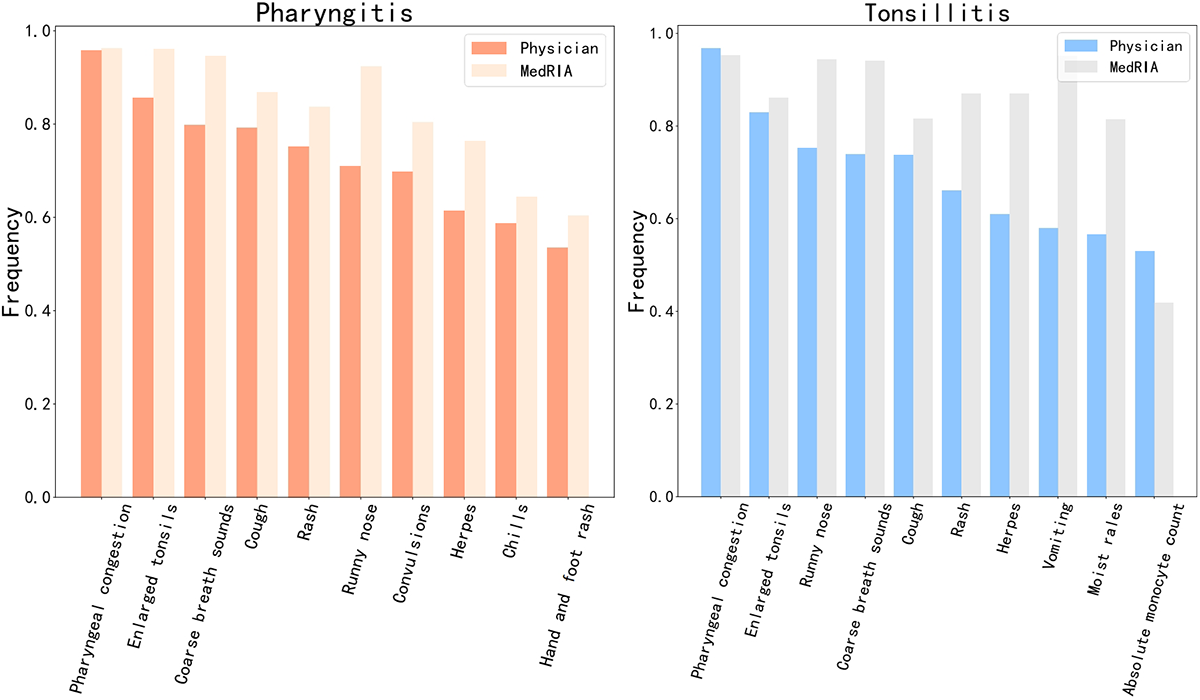

Supplement: Multimedia Appendix 6 [file jmir_v26i1e54616_app6.png]
