# Supplementary material for: AI-Driven Diagnostic Assistance in Medical Inquiry: Reinforcement Learning Algorithm Development and Validation
Source: J Med Internet Res. 2024 Aug 23;26:e54616. doi: 10.2196/54616 (PMC11380057; doi:10.2196/54616)

**Figure S3: Tree diagram of the inquiry features of MedRIA for four patients diagnosed with pharyngitis in the emergency task. These four patients shared similar characteristics, all being male with ages ranging from 31 to 32 years and complaining of sore throat. The numbers in brackets indicate the timesteps of inquiries.**


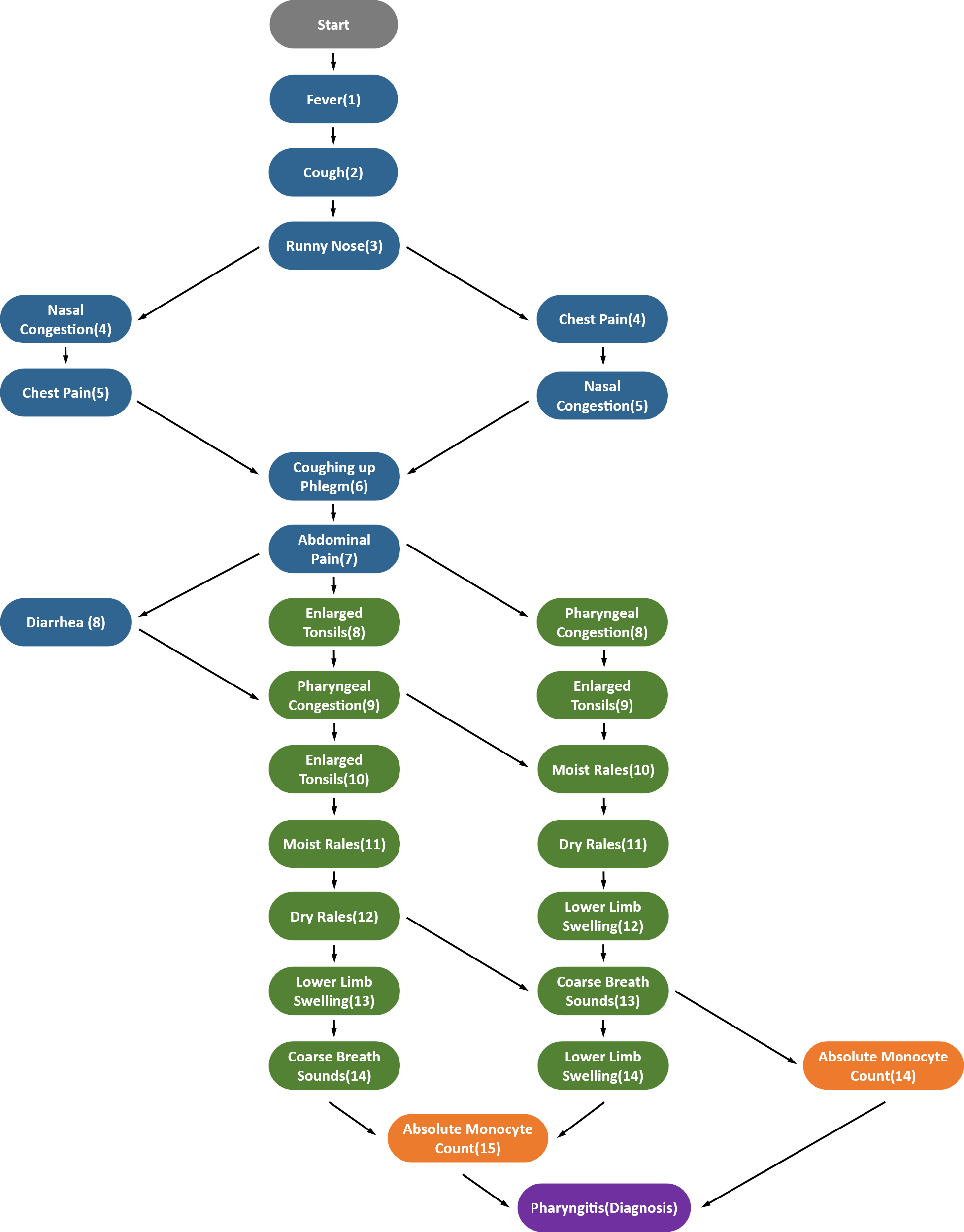

Supplement: Multimedia Appendix 7 [file jmir_v26i1e54616_app7.docx]
