# Supplementary material for: AI-Driven Diagnostic Assistance in Medical Inquiry: Reinforcement Learning Algorithm Development and Validation
Source: J Med Internet Res. 2024 Aug 23;26:e54616. doi: 10.2196/54616 (PMC11380057; doi:10.2196/54616)

# **Figure S4: Inquiry frequencies of the physician and MedRIA in the emergency task.**


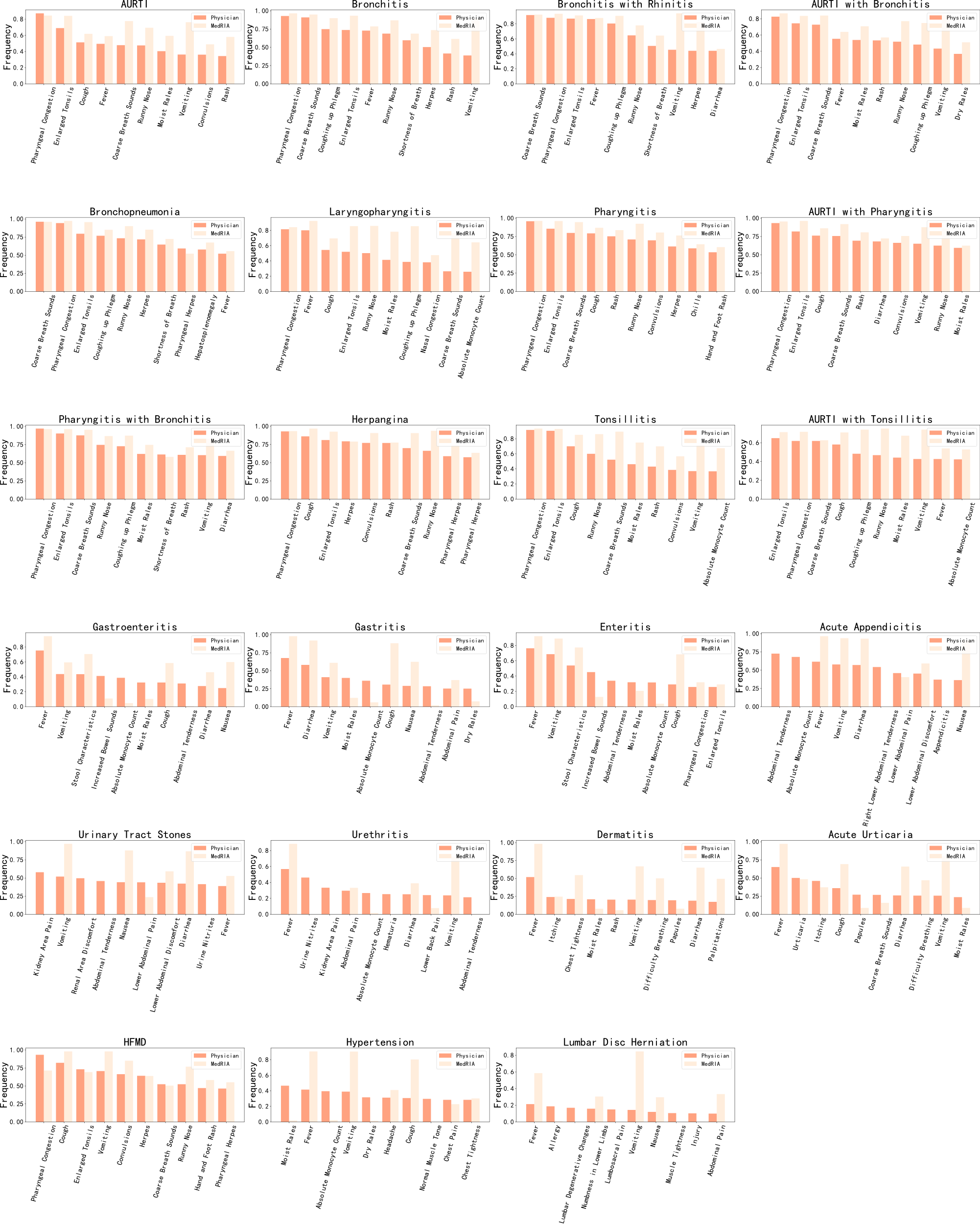


# **Figure S5: Inquiry frequencies of the physician and MedRIA in the pediatrics task.**


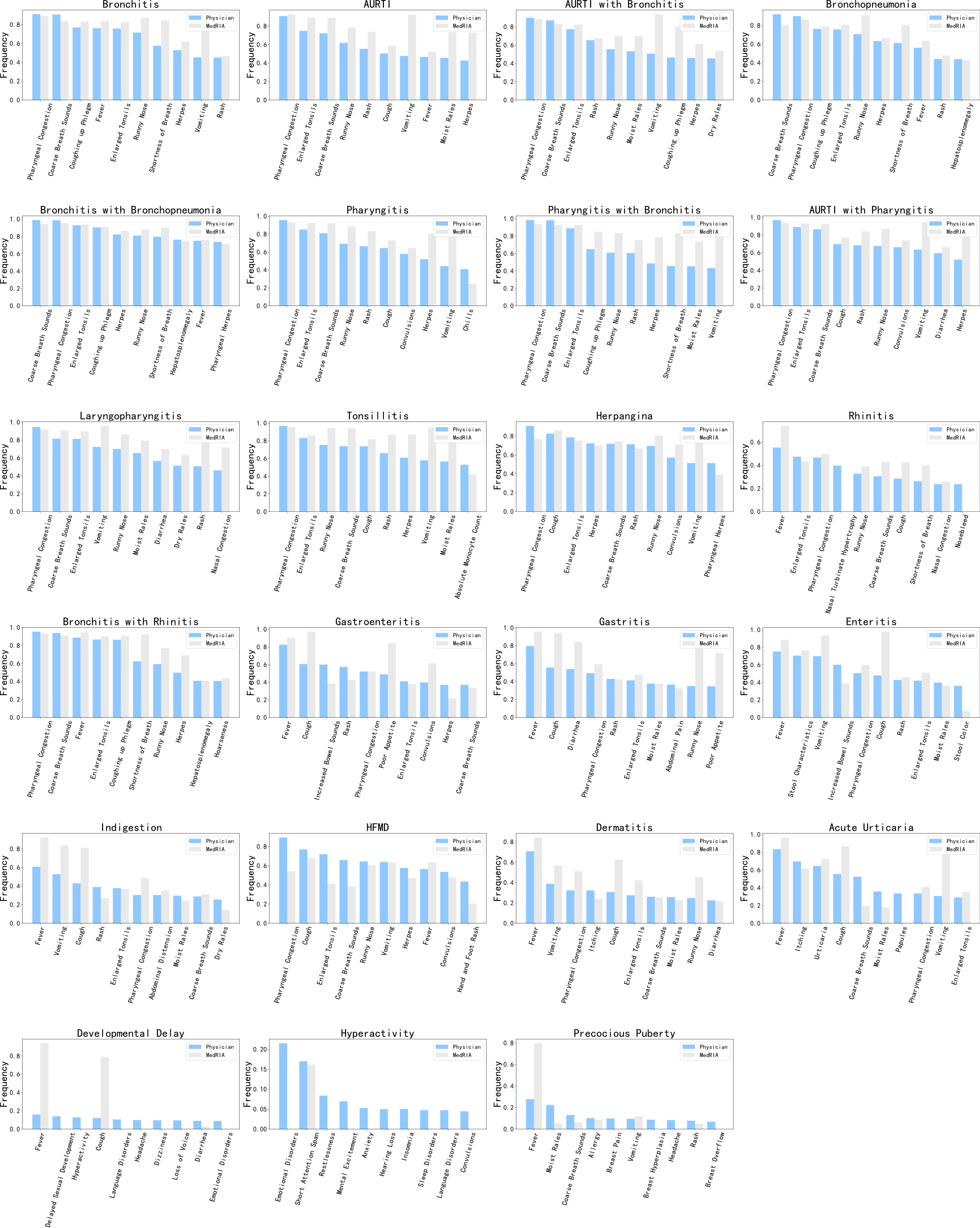


# **Figure S6: Inquiry heatmaps of MedRIA in the emergency task.**
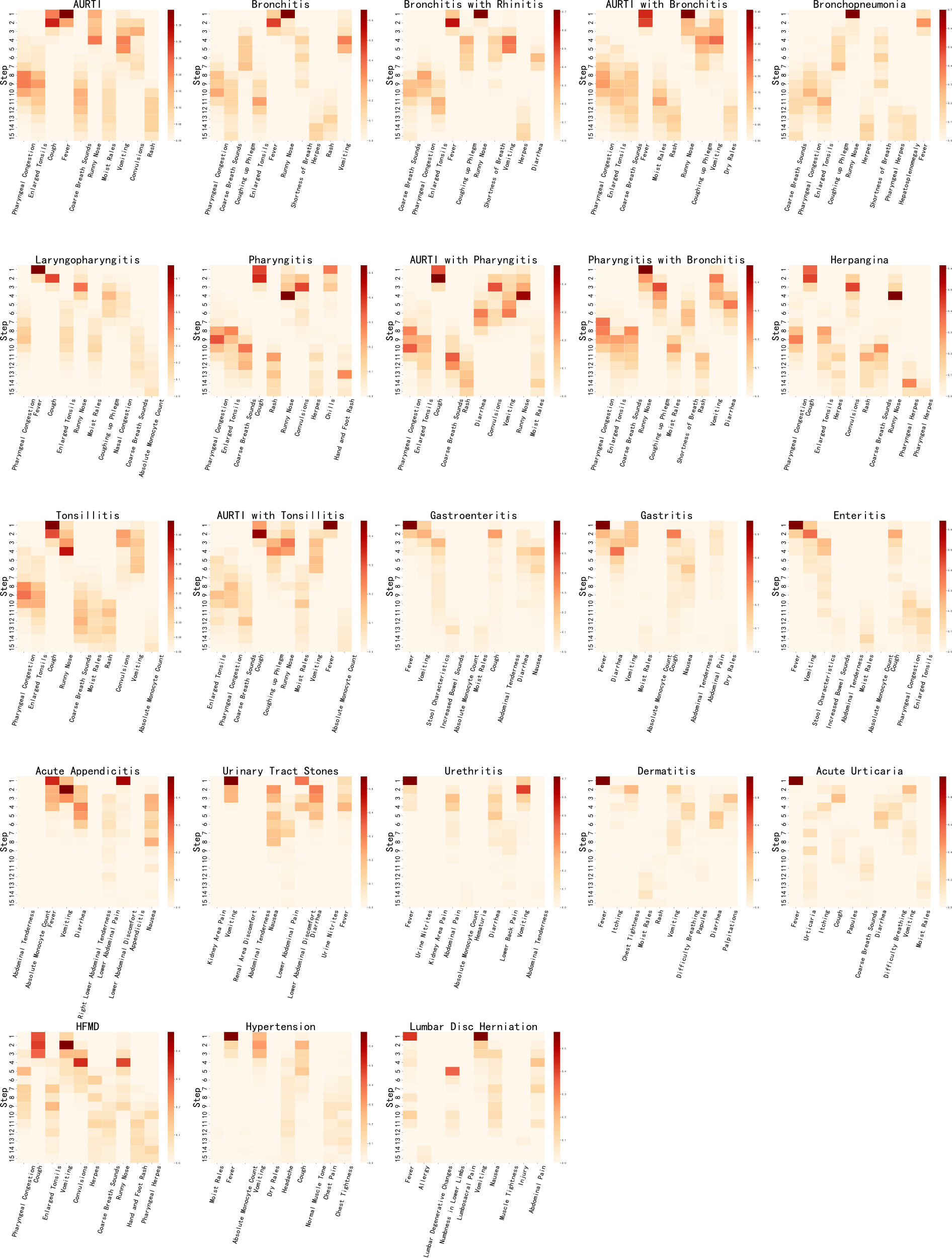


# **Figure S7: Inquiry heatmaps of the collaborative inquiry in the emergency task.**
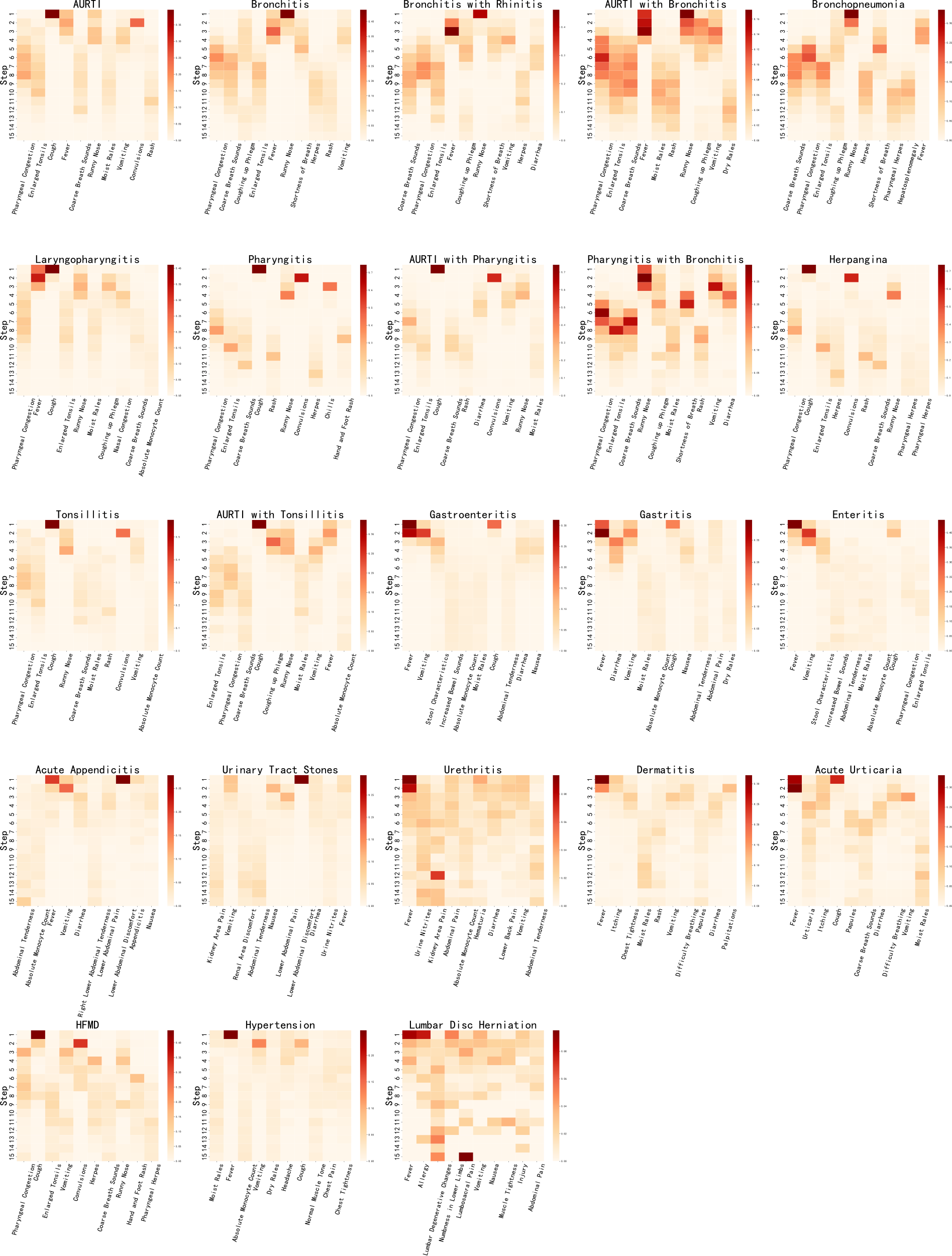


# **Figure S8: Inquiry heatmaps of MedRIA in the pediatrics task.**
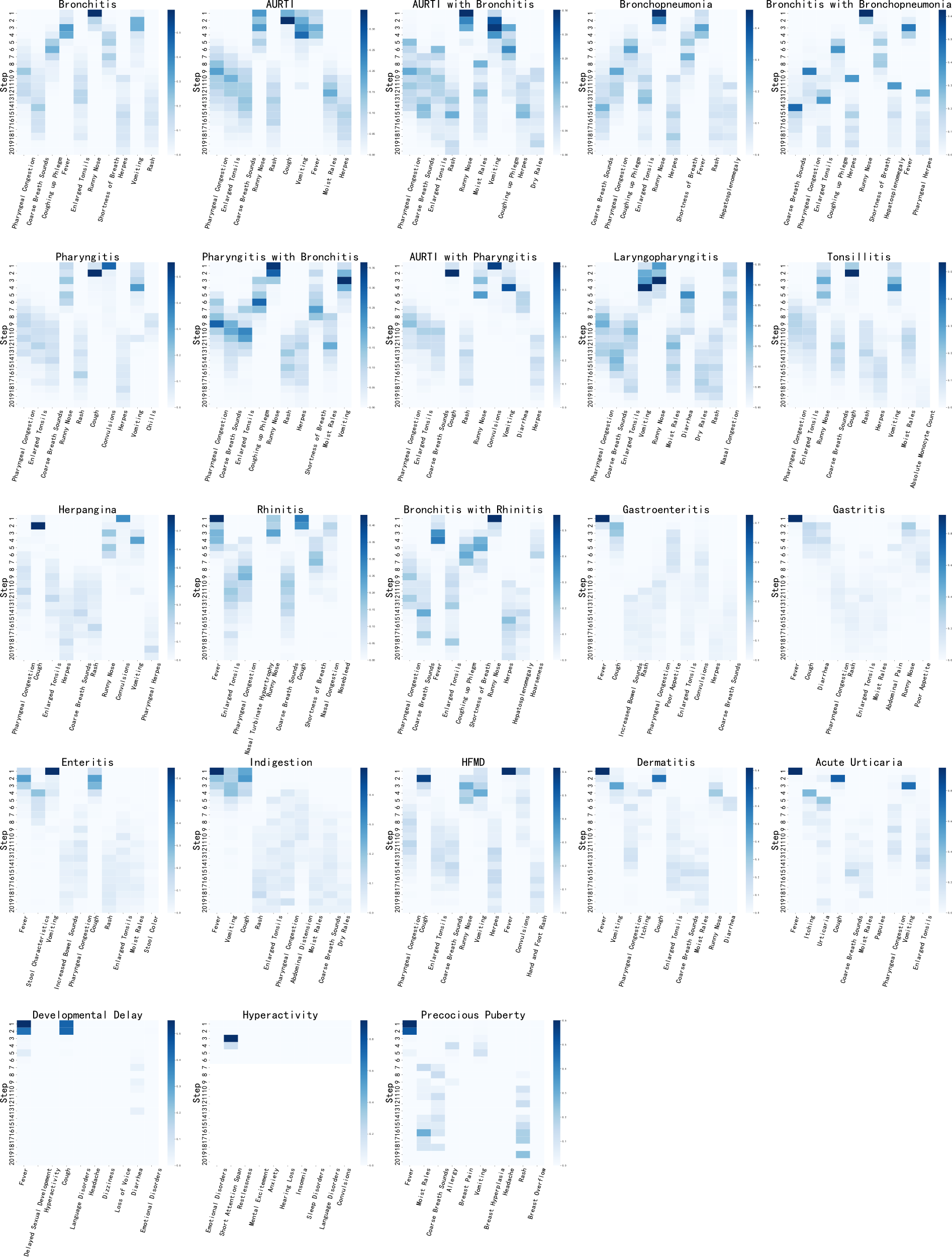


**Figure S9: Inquiry heatmaps of the collaborative inquiry in the pediatrics task.**
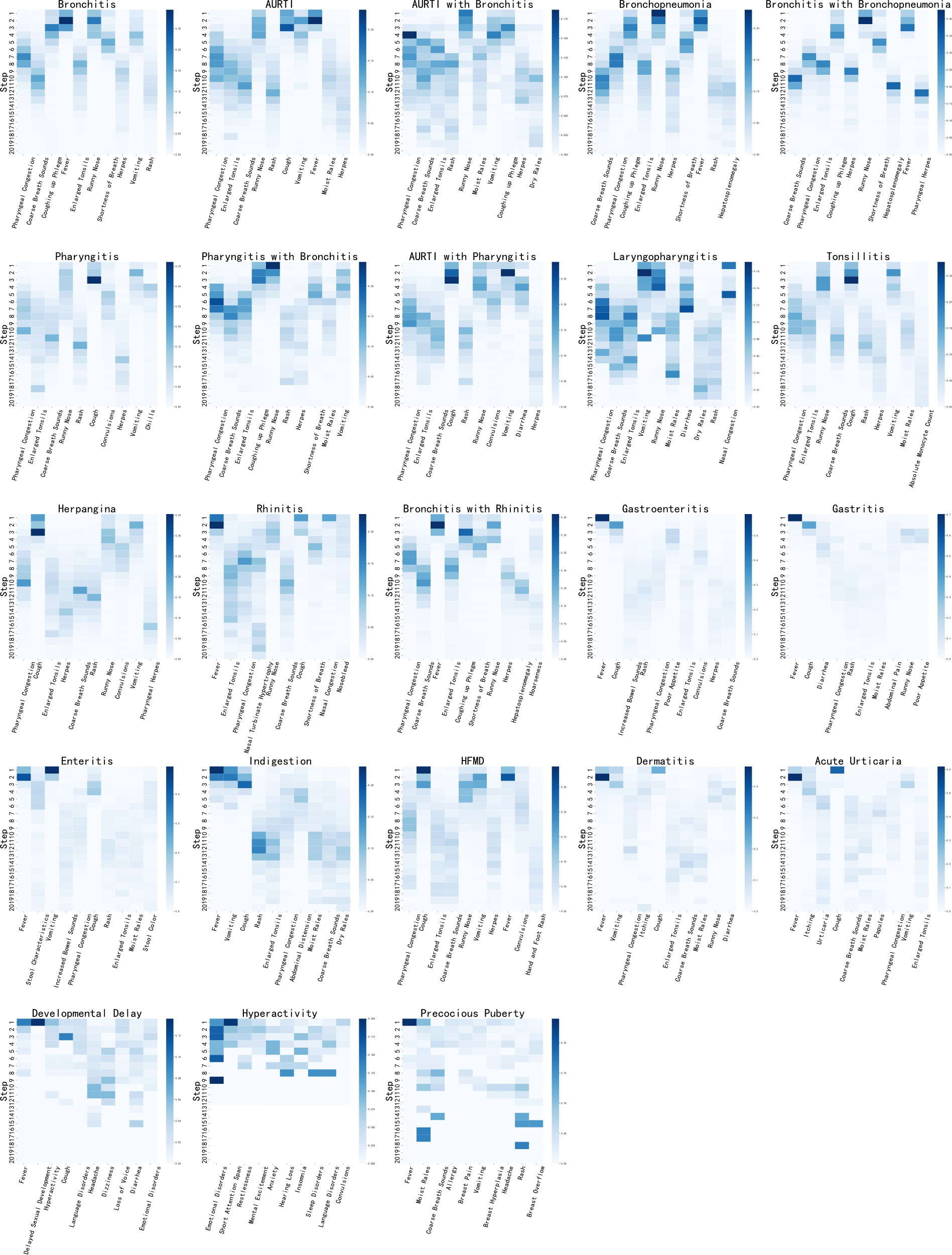

Supplement: Multimedia Appendix 8 [file jmir_v26i1e54616_app8.docx]
